# Native Coronary Artery Versus Saphenous Vein Graft Percutaneous Coronary Intervention in Patients with Prior Coronary Artery Bypass Grafting: An Updated Systematic Review and Meta-Analysis

**DOI:** 10.3390/jcm15218672

**Published:** 2026-11-06

**Authors:** Vaishvik Patel, Ahmed Al-Ghazawy, Jay Patel, Oluwakorede Akele, Ryan Englander, Abhishek Singh, Joseph Ingrassia, Khagendra Dahal

**Affiliations:** 1 Department of Internal Medicine, University of Connecticut Health, Farmington, CT 06030, USA; 2 Department of Cardiology, Hartford Hospital, Hartford, CT 06106, USA; 3 Division of Cardiology, University of Connecticut Health, Farmington, CT 06030, USA

**Keywords:** coronary artery bypass grafting, percutaneous coronary intervention, native coronary artery, saphenous vein graft, meta-analysis

## Abstract

**Background/Objectives:** Patients with prior coronary artery bypass grafting (CABG) frequently require repeat percutaneous coronary intervention (PCI), but the optimal target—native coronary artery or saphenous vein graft (SVG)—remains uncertain. We performed an updated systematic review and random-effects meta-analysis comparing clinical and procedural outcomes of native coronary artery PCI versus SVG PCI. **Methods:** PubMed, Embase, and the Cochrane Library (Cochrane Central Register of Controlled Trials) were searched from inception through May 2026. **Results:** Twenty-five studies (343,524 patients) were included, with 21 contributing to the MACE and/or all-cause mortality analyses. Compared with native-vessel PCI, SVG PCI was associated with higher rates of major adverse cardiovascular events (MACE) (risk ratio [RR] 1.30, 95% confidence interval [CI] 1.05–1.60; *p* = 0.017), all-cause mortality (RR 1.47, 95% CI 1.19–1.81; *p* < 0.001), myocardial infarction (RR 1.64, 95% CI 1.31–2.05; *p* < 0.001), in-stent restenosis (RR 1.71, 95% CI 1.19–2.44; *p* = 0.004), and target lesion revascularization (RR 1.70, 95% CI 1.16–2.47; *p* = 0.006). After exclusion of the single randomized trial, associations for MACE (RR 1.38, 95% CI 1.12–1.71) and all-cause mortality (RR 1.50, 95% CI 1.21–1.85) were slightly stronger. Cardiac death, stroke, and bleeding did not differ significantly. Findings for MACE and target lesion revascularization were sensitive to the omission of single studies. In the exploratory drug-eluting stent-predominant subgroup, effect directions were generally consistent. Despite these associations, most included studies were observational, and substantial heterogeneity and residual confounding remain important limitations (95% prediction intervals for MACE and mortality included the null), and the certainty of evidence (GRADE) was low to very low. **Conclusions:** SVG PCI was associated with less favorable long-term outcomes than native coronary artery PCI, although these findings do not establish causality and should be interpreted alongside randomized evidence supporting individualized, anatomy-based target selection. Adequately powered randomized trials with extended follow-up are needed to define the optimal revascularization strategy across the spectrum of post-CABG anatomy.

## 1. Introduction

Patients with prior coronary artery bypass grafting (CABG) frequently require repeat coronary revascularization because of progressive native coronary atherosclerosis and late bypass graft failure. Saphenous vein grafts (SVGs), used in approximately 95% of CABG procedures, have limited long-term durability, with failure rates approaching 60% beyond 10 years [1,2]. Consequently, repeat percutaneous coronary intervention (PCI) is commonly performed, targeting either the native coronary artery or a degenerated SVG. Although approximately two-thirds of repeat PCI procedures target native coronary arteries, the optimal target vessel remains uncertain [1,2,3,4,5].

Observational studies have consistently reported more favorable procedural and long-term outcomes with native coronary artery PCI than SVG PCI, including lower rates of myocardial infarction (MI), repeat revascularization, major adverse cardiovascular events (MACE), and mortality. These findings have informed contemporary practice and guidelines, which generally favor native-vessel PCI when technically feasible. However, observational comparisons are susceptible to confounding by indication, as native-vessel PCI is often avoided in the presence of chronic total occlusion (CTO), diffuse disease, or unfavorable anatomy, whereas SVG PCI may be selected when the native vessel is technically unsuitable.

The recently published PROCTOR trial is the latest randomized trial to compare native coronary artery PCI versus SVG PCI in patients with coronary artery bypass grafting (CABG). In contrast to observational evidence, PROCTOR demonstrated lower 1-year MACE with SVG PCI, driven primarily by fewer periprocedural myocardial infarction (MI) events and target coronary territory revascularizations. However, the trial was terminated early after enrolling 220 of 584 planned patients because of slow recruitment and challenges with enrollment and included only patients in whom a multidisciplinary Heart Team deemed both targets technically feasible and clinically appropriate. The native-vessel arm also had a greater burden of CTOs and complex anatomy, potentially contributing to procedural risk. Clinical presentation may further influence outcomes, as available studies include mixed acute coronary syndrome (ACS) and chronic coronary syndrome (CCS) populations; SVG PCI in ACS carries particular risks of distal embolization, no-reflow, and periprocedural MI, whereas CCS permits greater time for anatomical assessment and complex native-vessel PCI [6,7].

The observational and randomized evidence should be viewed as complementary rather than inherently discrepant because the studies address different patient populations, anatomical constraints, and time horizons. We therefore performed an updated systematic review and meta-analysis comparing native coronary artery PCI with SVG PCI, incorporating contemporary observational studies and the recently published randomized evidence. Our objective was to compare long-term clinical outcomes, repeat revascularization, and procedural characteristics and to further define the role of native-vessel versus SVG PCI after CABG.

## 2. Methods

### 2.1. Search Strategy and Study Selection

A systematic search of three electronic databases—PubMed, Embase, and the Cochrane Library (Cochrane Central Register of Controlled Trials)—was conducted from database inception to identify studies comparing native coronary artery PCI with SVG PCI in patients with prior CABG. The date of the last search was May 2026 for PubMed, Embase, and the Cochrane Library. The complete search strategy for each database, including Boolean operators, MeSH and Emtree terms, truncation, and filters, is provided in Supplementary Methods S1. The search strategy employed combinations of terms including “percutaneous coronary intervention,” “saphenous vein graft,” “native coronary artery,” “coronary artery bypass grafting,” and related synonyms. No language restrictions were applied. The review was conducted in accordance with the Preferred Reporting Items for Systematic Reviews and Meta-Analyses (PRISMA) guidelines [8]. This systematic review and meta-analysis was prospectively registered with the International Prospective Register of Systematic Reviews (PROSPERO; registration number: CRD420261449018). The registered protocol is publicly accessible in the PROSPERO database. The following amendments were made after registration, during peer review, and are reported as deviations from the protocol: (1) all pooled analyses to which the randomized trial contributed were repeated after its exclusion; (2) 95% prediction intervals and Hartung–Knapp–Sidik–Jonkman-adjusted confidence intervals were added; and (3) the certainty of the evidence was graded using GRADE. Study selection was performed independently by two reviewers at the title/abstract and full-text stages, with disagreements resolved by consensus or adjudication by a third reviewer. No automation tools were used.

Studies were grouped for synthesis according to clinical outcomes (MACE, mortality, MI, ISR, cardiac death, stroke, bleeding, TLR, and TVR) and procedural characteristics (stent diameter, stent length, lesion length, and number of stents).

Studies were eligible for inclusion if they: (1) enrolled patients with CABG undergoing PCI; (2) compared outcomes between native coronary artery PCI and SVG PCI; and (3) reported at least one clinical outcome of interest. Both randomized controlled trials and observational studies (prospective and retrospective) were included. Case reports, editorials, reviews without original data, and studies with fewer than 50 patients were excluded. For the in-stent restenosis analysis, only studies in which stents were implanted in the treated lesions were eligible; studies reporting restenosis after balloon angioplasty without stent implantation were excluded from this outcome because restenosis after balloon angioplasty and in-stent restenosis differ in mechanism and definition.

For each outcome, we sought the longest available follow-up reported by each study and extracted the available compatible effect estimate or arm-level event data. Variables sought also included study design, sample size, demographics, comorbidities, clinical presentation, prior CABG characteristics, lesion/graft characteristics, procedural features, stent type, and follow-up duration. When information was not reported, it was treated as unavailable rather than imputed.

### 2.2. Data Extraction and Outcomes

Studies were included in each synthesis only when they reported the corresponding outcome and provided data that could be expressed as a common effect measure. Individual-study results were tabulated in the study-level tables and displayed graphically using forest plots; funnel plots were used for assessment of dissemination/publication bias where sufficient studies were available. Continuous outcomes were synthesized as mean differences and binary outcomes as risk ratios.

Two independent reviewers extracted data using a standardized form. Discrepancies were resolved by consensus or adjudication by a third reviewer. Extracted variables included study design, sample size, patient demographics, comorbidities, procedural characteristics, stent type, and clinical outcomes at the longest available follow-up. No study investigators were contacted for additional data. When reported summary statistics were incomplete, the prespecified conversion method described below was used; otherwise, the outcome was not extracted.

Risk of bias due to missing results was assessed using funnel plots and Egger’s test when the number of contributing studies was sufficient; these methods were interpreted as assessments of possible dissemination/publication bias rather than substitutes for study-level risk-of-bias assessment.

Potential clinical and methodological sources of heterogeneity were explored descriptively through the prespecified DES-predominant subgroup analysis and by examining study design, follow-up duration, clinical presentation, and anatomical/procedural differences across contributing studies. No meta-regression was performed because the available number of studies and completeness of study-level covariates were insufficient.

Certainty of evidence was assessed with the GRADE approach, as described in Section 2.5.

The major clinical outcomes included MACE, all-cause mortality, MI, in-stent restenosis (ISR), cardiac death, stroke, bleeding, target lesion revascularization (TLR), and target vessel revascularization (TVR). Procedural outcomes included stent diameter, stent length, lesion length, and number of implanted stents.

### 2.3. Statistical Analysis

**Figure 1:**
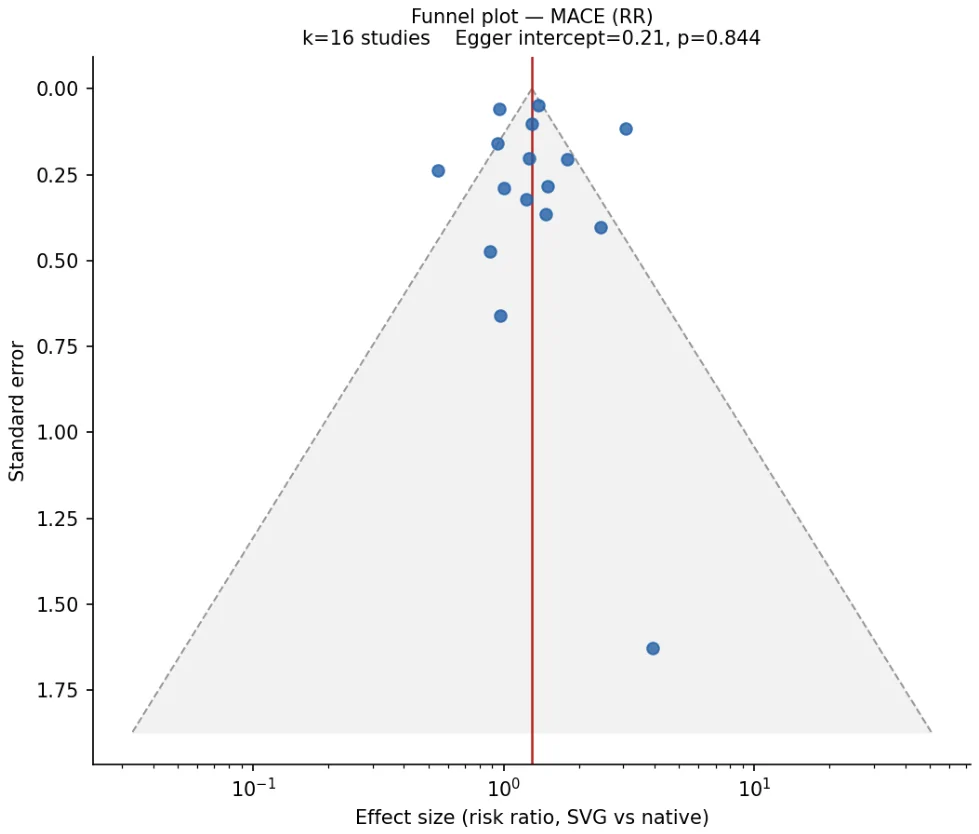
Funnel plot assessing publication bias for major adverse cardiac events (MACE) in SVG PCI versus native coronary artery PCI.

**Figure 2:**
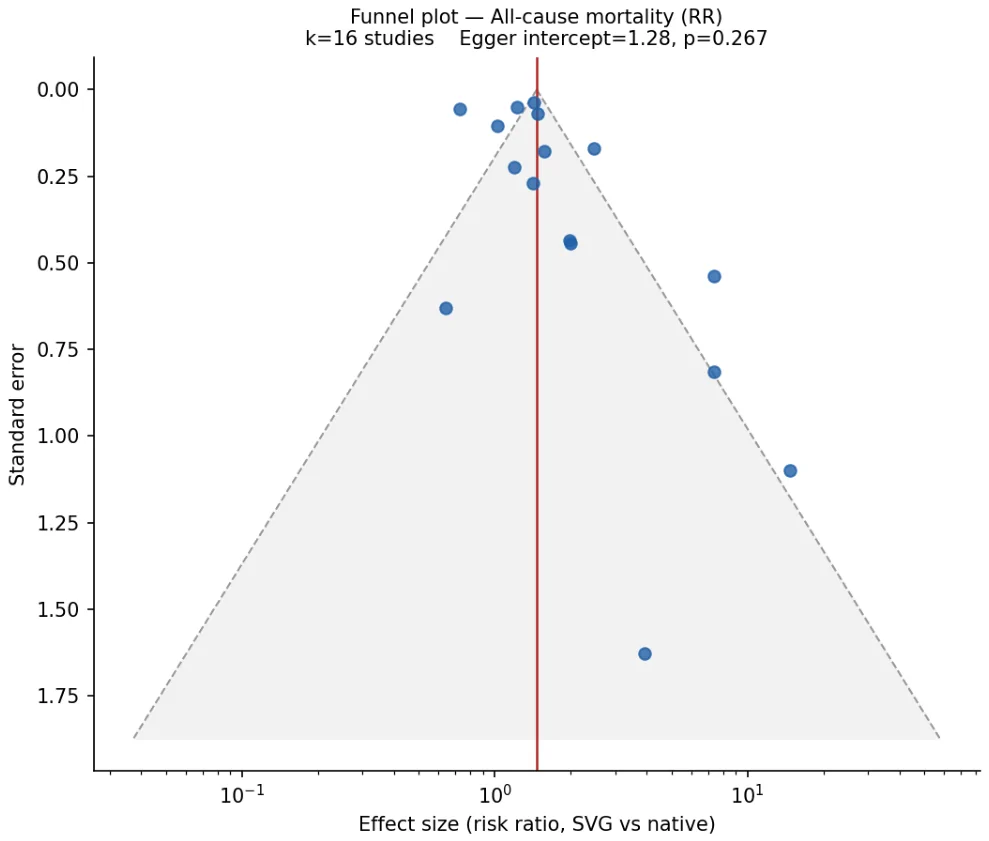
Funnel plot assessing publication bias for all-cause mortality in SVG PCI versus native coronary artery PCI.

Random-effects meta-analyses using the DerSimonian-Laird method were performed to estimate pooled risk ratios (RRs) for binary outcomes and mean differences (MDs) for continuous outcomes, with 95% confidence intervals (CIs). For studies reporting medians and interquartile ranges, means and standard deviations were estimated using the method of Wan et al. (2014) [9]. Heterogeneity was assessed using the I^2^ statistic and Cochran’s Q test. Because I^2^ quantifies the proportion of variability attributable to between-study differences but not its magnitude, between-study variance (τ^2^) was also estimated using the DerSimonian–Laird estimator, and 95% prediction intervals, which describe the range of effects expected in a new comparable setting, were calculated for pooled estimates with at least three studies using a t-distribution with k−2 degrees of freedom [10,11]. As a sensitivity analysis, confidence intervals were recalculated with the Hartung–Knapp–Sidik–Jonkman adjustment, which accounts for uncertainty in the estimated between-study variance. Publication bias was evaluated using funnel plots and Egger’s test where applicable; funnel plots were generated for outcomes with a sufficient number of contributing studies and are presented in Figure 1 and Figure 2.

Leave-one-out sensitivity analyses were performed for all pooled outcomes to assess the robustness of findings. Each analysis re-pooled the outcome after sequentially dropping one study at a time. Results were classified as “robust” if the pooled estimate remained statistically significant in every omission, “fragile” if significance was lost in at least one omission, and “not significant” if the full model was not significant. The evidence base comprised one randomized trial (PROCTOR) and 24 nonrandomized studies. In the primary analyses, PROCTOR was pooled with the nonrandomized studies for each outcome to which it contributed, because all studies addressed the same comparison. Because randomized and nonrandomized studies are susceptible to different biases, and because PROCTOR showed an effect direction opposite to that of most observational studies, all pooled analyses to which PROCTOR contributed were repeated after its exclusion (nonrandomized studies only), and the PROCTOR estimate is reported separately. This sensitivity analysis was not prespecified in the PROSPERO protocol and was added during peer review.

An exploratory subgroup analysis was performed according to DES predominance, with studies classified by the proportion of DES use in the native-vessel arm (≥0.5 = DES-predominant). Given that the evidence base was overwhelmingly DES-predominant (10 DES-predominant vs. 2 BMS/mixed studies with stent-type data; 13 studies had no stent-type data), between-group comparisons were underpowered and should be interpreted as descriptive only.

### 2.4. Risk of Bias Assessment

Risk of bias was assessed at the level of each outcome estimate, and domain-level and overall judgments for each included study are presented in Supplementary Table S5 (ROBINS-I, 24 nonrandomized studies, with the basis for each judgment) and S6 (RoB 2, PROCTOR trial). The PROCTOR randomized trial was evaluated using the Cochrane Risk of Bias 2 (RoB 2) tool (22 August 2019 version) for the effect of assignment to intervention. The 5 RoB 2 domains addressed the randomization process, deviations from intended interventions, missing outcome data, measurement of the outcome, and selection of the reported result. Nonrandomized studies were evaluated using the Risk of Bias in Non-randomized Studies of Interventions (ROBINS-I; 2016 version) [12], across the domains of confounding, selection of participants, classification of interventions, deviations from intended interventions, missing data, outcome measurement, and selection of the reported result.

For the nonrandomized evidence, the target effect was the effect of initiating native-coronary PCI rather than SVG PCI at the index procedure. Prespecified confounders included age, diabetes, renal disease, left ventricular dysfunction or heart failure, previous myocardial infarction and PCI, clinical presentation, cardiogenic shock, time since CABG or graft age, native-vessel chronic total occlusion and lesion complexity, graft degeneration or thrombus burden, myocardium at risk, and technical feasibility of each target. Procedural success, post-PCI flow, no-reflow, final stent length, and length of stay were considered post-intervention variables and were not accepted as substitutes for adequate control of baseline confounding.

We categorized RoB 2 judgments as low risk, some concerns, or high risk. ROBINS-I judgments were categorized as low, moderate, serious, critical, or no information [13]. Overall judgments followed the domain-based rules of the respective tools and were not converted to numerical quality scores. We interpreted randomized and nonrandomized evidence separately and therefore reported all pooled clinical estimates both with and without the randomized trial (Section 2.3).

We used risk-of-bias judgments to qualify causal interpretation. Because no included estimate was judged at critical risk of bias and all nonrandomized estimates were judged at serious risk, sensitivity analyses restricted by risk-of-bias category were not feasible; the analysis excluding the randomized trial (Section 2.3) was performed instead. We treated funnel plots and small-study-effect tests as assessments of dissemination bias rather than substitutes for study-level risk-of-bias assessment.

### 2.5. Certainty of the Evidence

The certainty of the evidence for each clinical outcome was assessed using the Grading of Recommendations Assessment, Development and Evaluation (GRADE) approach [14,15]. The assessment was applied to the primary pooled analyses, based on the 25 included studies, including the PROCTOR trial. Because nonrandomized studies predominate (15 of 16 studies for MACE and mortality; PROCTOR carries about 7% of the weight or less), the pooled body of evidence was rated as nonrandomized evidence. Two further assessments were made: a sensitivity analysis pooling the nonrandomized studies without PROCTOR, and a separate rating of PROCTOR as randomized evidence. Because risk of bias in nonrandomized studies was appraised with ROBINS-I, the nonrandomized evidence started at high certainty and was rated down for risk of bias (all nonrandomized studies were judged at serious overall risk, principally because of confounding), inconsistency (I^2^, between-study variance, direction of effects, and whether the 95% prediction interval included no effect), indirectness (population, follow-up horizon, and outcome definition), imprecision (width of the 95% confidence interval relative to no effect and to appreciable harm, and optimal information size), and publication bias (funnel plots and Egger’s test when at least 10 studies contributed). Rating up for a large effect (RR > 2 or <0.5), dose–response, or opposing residual confounding was considered but not applied when serious risk-of-bias concerns remained. PROCTOR started at high certainty and was rated using the RoB 2 judgments. Certainty was categorized as high, moderate, low, or very low. Absolute effects were estimated by applying the pooled RR to the median native-vessel event rate across studies (pooled analyses) or to the control-arm event rate (PROCTOR) and are approximate because follow-up ranged from 30 days to 5 years.

## 3. Results

### 3.1. Study Characteristics

**Figure 3:**
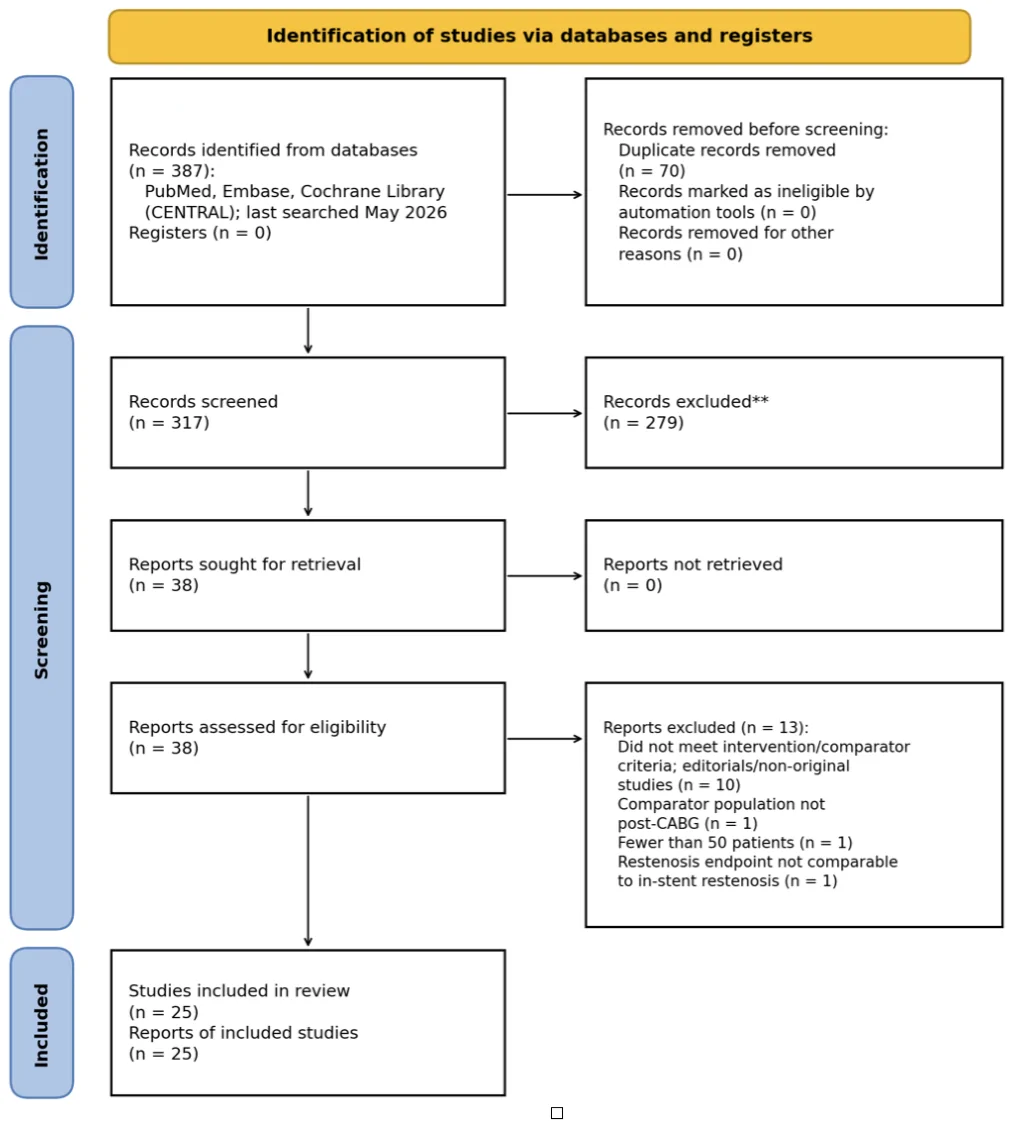
PRISMA 2020 flow diagram of study selection. ** Records excluded at title and abstract screening by two independent reviewers; no automation tools were used.

Twenty-five studies (24 nonrandomized studies and one randomized trial; 343,524 unique patients) met overall inclusion criteria for this meta-analysis comparing PCI of native coronary vessels versus SVGs in post-CABG patients; of these, 21 studies reported data suitable for pooling MACE and/or all-cause mortality (16 studies for each outcome) at the longest available follow-up, with the remaining studies contributing to secondary clinical and continuous endpoints. The study-selection process is summarized in the PRISMA 2020 flow diagram (Figure 3): of 387 records identified, 70 duplicates were removed, 317 records were screened, and 38 full-text reports were assessed; 13 reports were excluded, including Waldo et al., 2013 [16] (comparator population not post-CABG), Meliga et al., 2007 [17] (fewer than 50 patients), and Ernst et al., 1987 [18] (balloon-angioplasty restenosis in the pre-stent era, not comparable to in-stent restenosis). The design and setting of each included study are listed in Table 1 and Table 2, and all included studies are cited [5,7,19,20,21,22,23,24,25,26,27,28,29,30,31,32,33,34,35,36,37,38,39,40,41].

The updated search incorporated contemporary observational evidence published after earlier meta-analyses, as well as the PROCTOR randomized trial. Baseline comparability was reasonable across arms: absolute differences in reported risk-factor prevalence between native and SVG cohorts were modest (all |Δ| ≤ 6 percentage points; largest for prior PCI [Δ = 0.060] and PVD [Δ = 0.058]), though data availability was inconsistent across studies (k = 3–9 per variable). Table 1 and Table 2 present study-level estimates for the MACE and all-cause mortality analyses, respectively.

### 3.2. Primary Efficacy and Safety Outcomes

**Figure 4:**
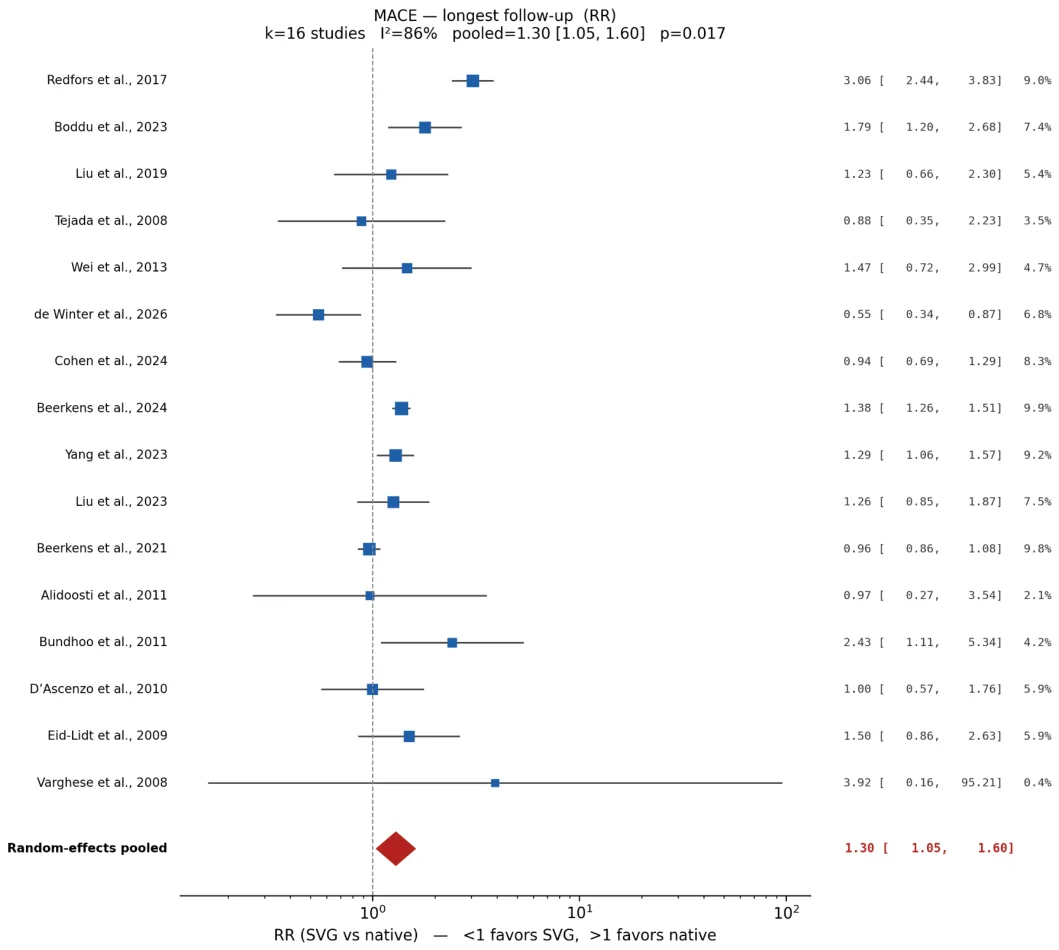
Forest plot of major adverse cardiac events (MACE) comparing SVG PCI with native coronary artery PCI (random-effects meta-analysis) [7,20,21,22,24,25,30,31,32,33,34,36,37,38,39,40].

**Figure 5:**
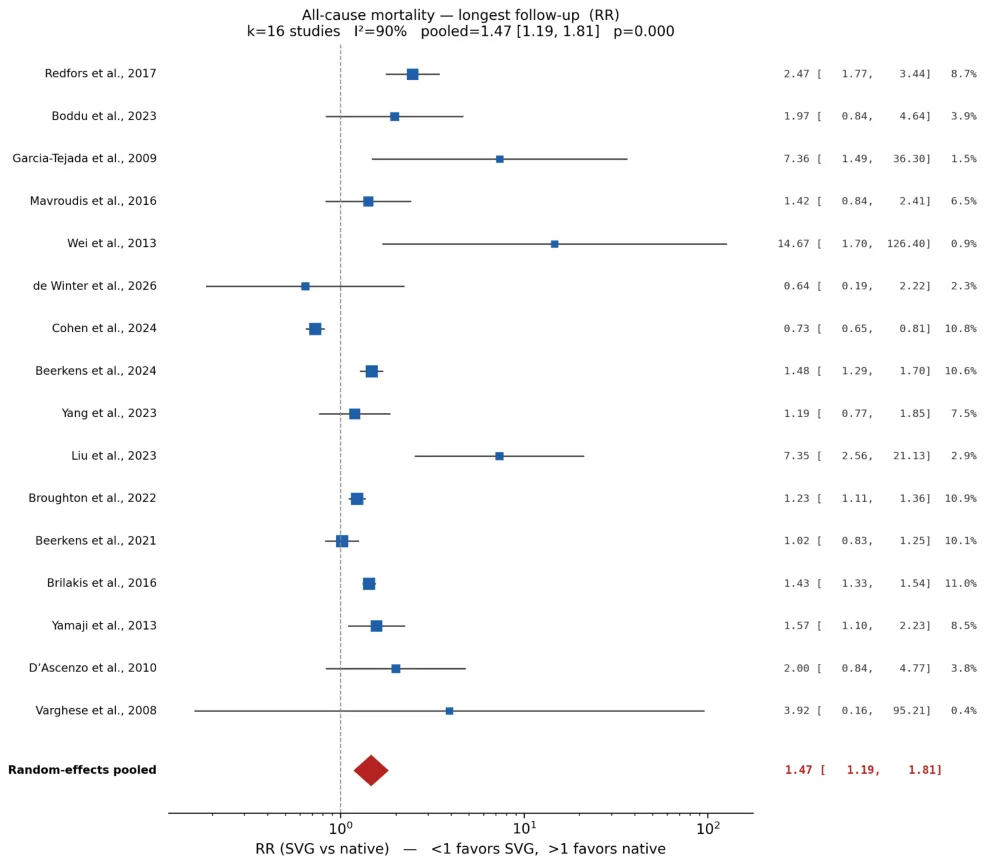
Forest plot of all-cause mortality comparing SVG PCI with native coronary artery PCI (random-effects meta-analysis) [5,7,21,22,27,28,29,31,32,33,34,37,38,39,40,41].

**Figure 6:**
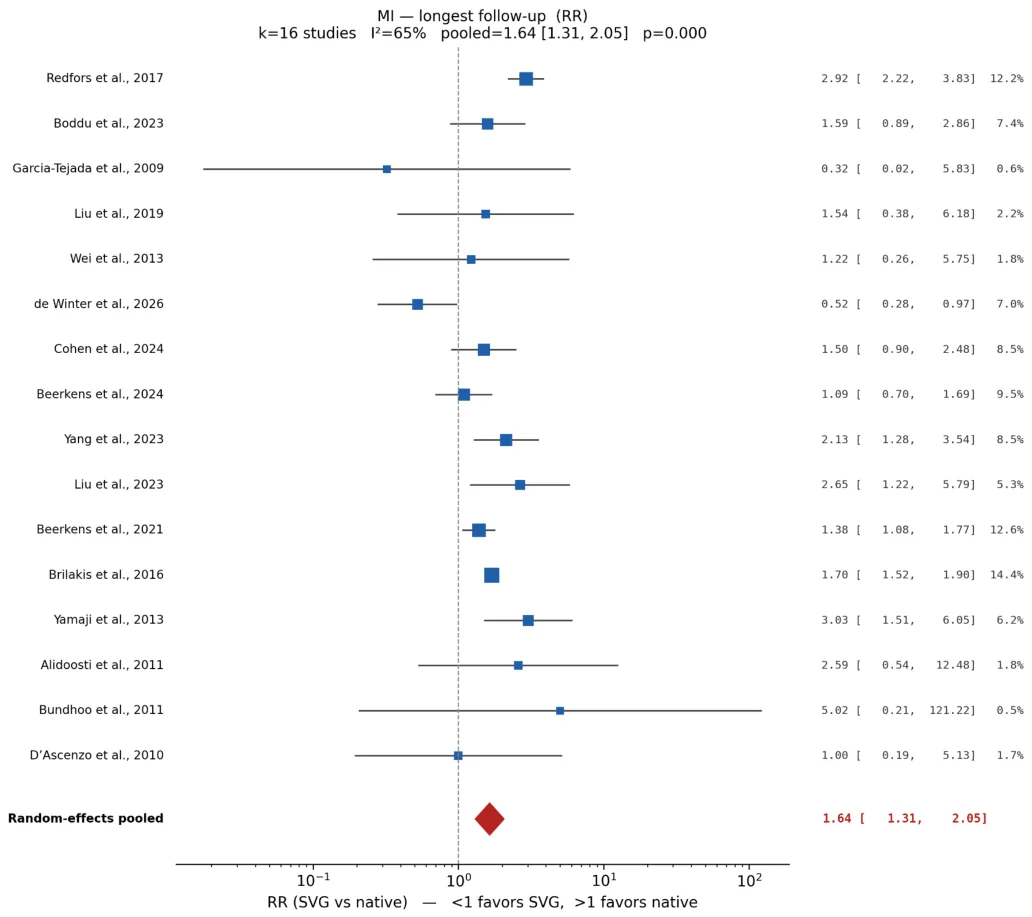
Forest plot of myocardial infarction comparing SVG PCI with native coronary artery PCI (random-effects meta-analysis) [5,7,21,24,25,27,28,30,31,32,33,34,37,38,39,40].

**Figure 7:**
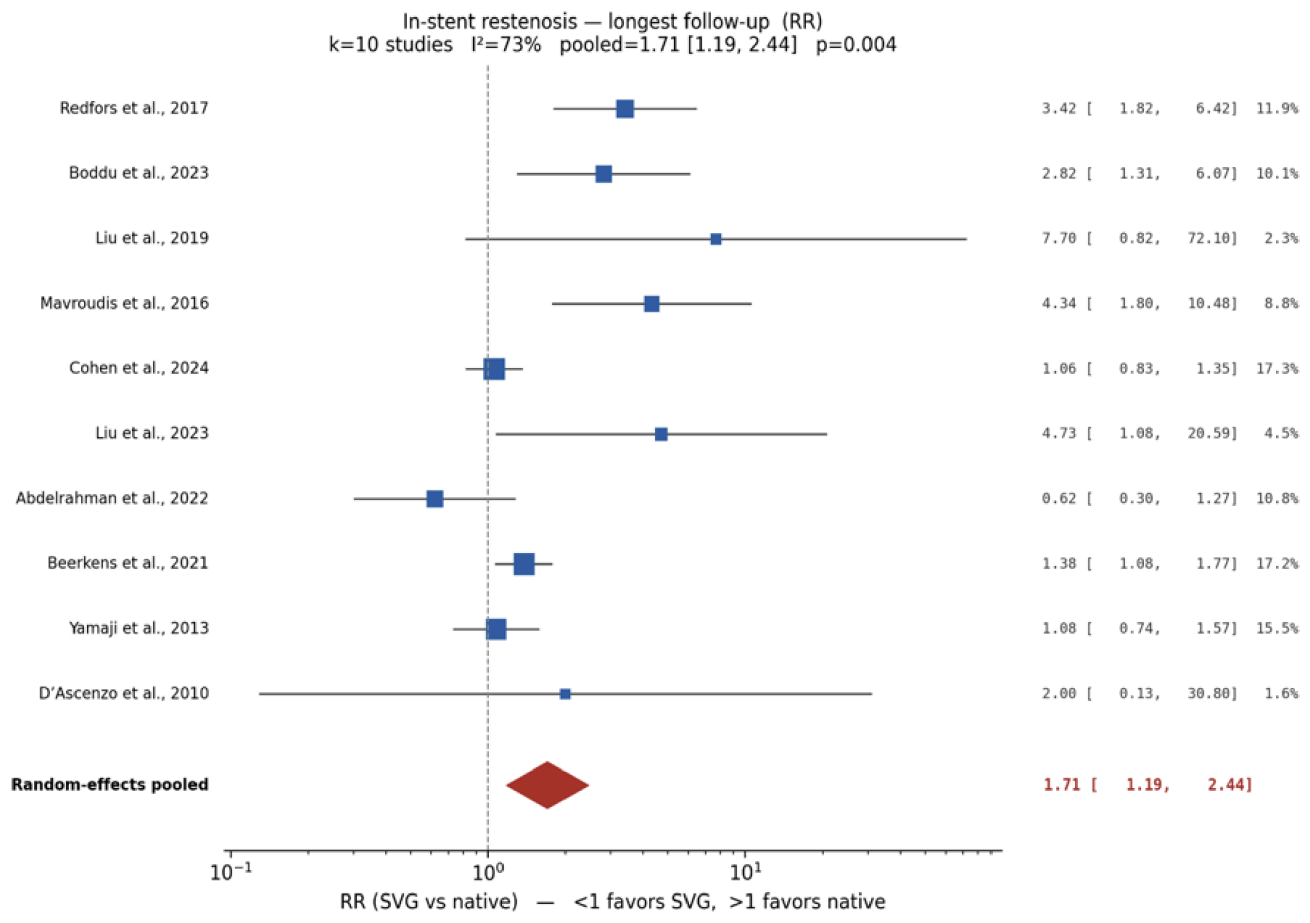
Forest plot of in-stent restenosis comparing SVG PCI with native coronary artery PCI (random-effects meta-analysis) [19,21,27,29,30,31,34,37,38,39].

Among the 21 studies contributing to MACE and/or all-cause mortality analyses, random-effects pooling showed that SVG PCI was associated with significantly higher rates of MACE (RR 1.30, 95% CI 1.05–1.60; *p* = 0.017; I^2^ = 86%), all-cause mortality (RR 1.47, 95% CI 1.19–1.81; *p* < 0.001; I^2^ = 90%), MI (RR 1.64, 95% CI 1.31–2.05; *p* < 0.001; I^2^ = 65%; k = 16), and ISR (RR 1.71, 95% CI 1.19–2.44; *p* = 0.004; I^2^ = 73%; k = 10) relative to native-vessel PCI. Forest plots of the major outcomes are shown in Figure 4, Figure 5, Figure 6 and Figure 7, with study-level estimates for MACE and all-cause mortality detailed in Table 1 and Table 2, respectively.

**Figure 8:**
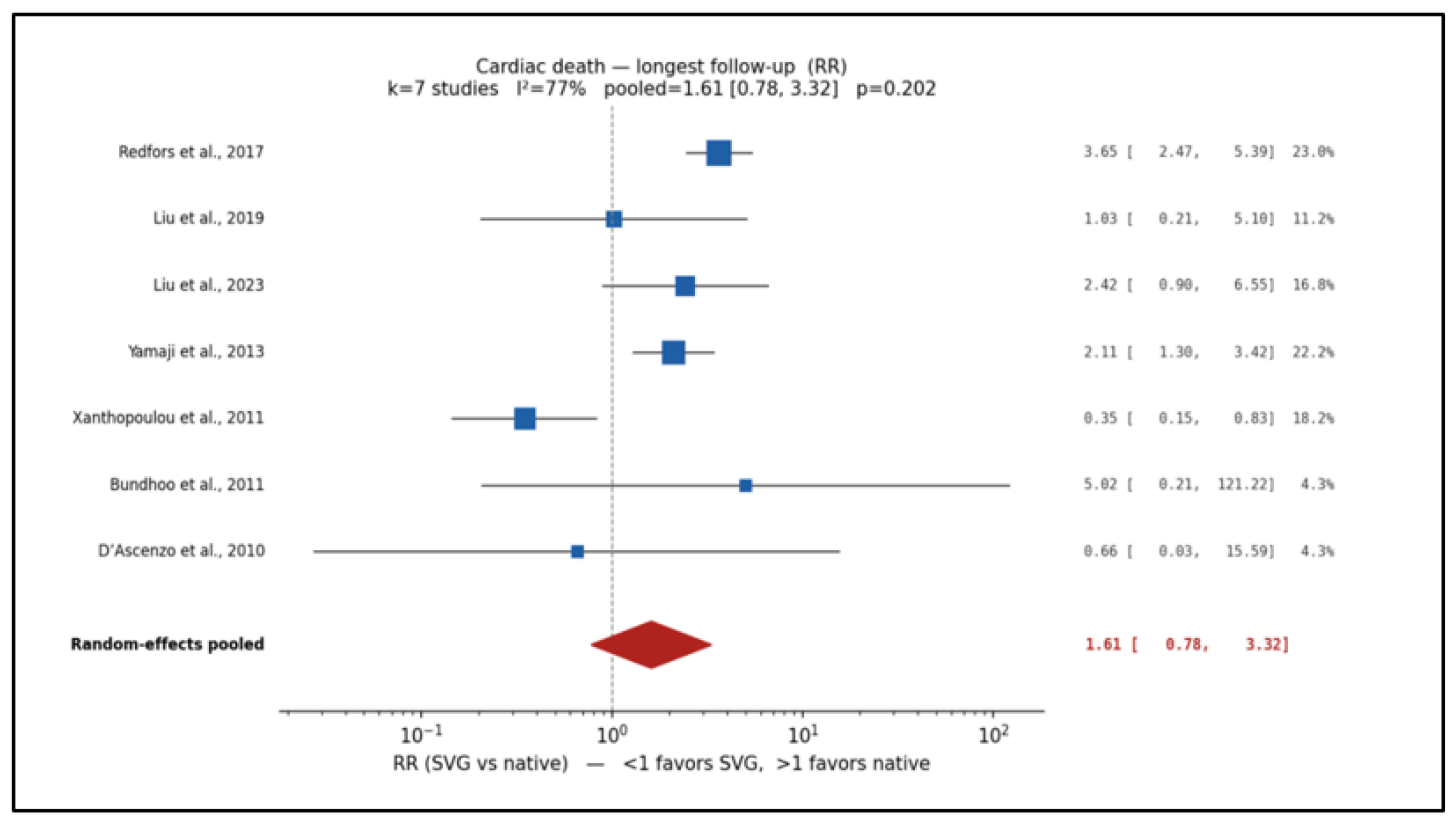
Forest plot of cardiac death comparing SVG PCI with native coronary artery PCI (random-effects meta-analysis) [21,24,26,27,30,37,39].

**Figure 9:**
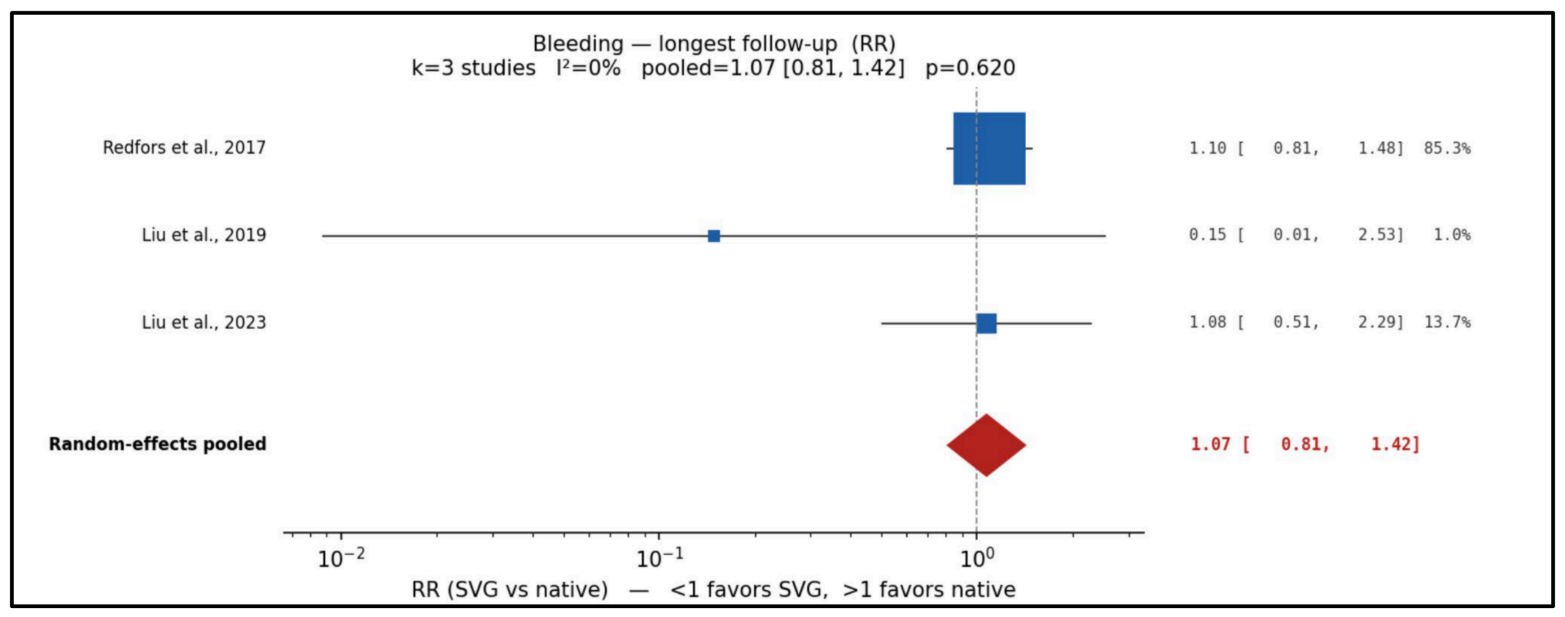
Forest plot of bleeding complications comparing SVG PCI with native coronary artery PCI (random-effects meta-analysis) [30,37,39].

Cardiac death (RR 1.61, 95% CI 0.78–3.32; k = 7) and stroke (RR 0.94, 95% CI 0.45–1.98; k = 4) did not differ significantly, and bleeding was similar between strategies (RR 1.07, 95% CI 0.81–1.42; k = 3; I^2^ = 0%). Forest plots for these additional clinical outcomes are included in Figure 8 and Figure 9.

Given the high between-study heterogeneity, 95% prediction intervals were calculated. For MACE, the between-study standard deviation was τ = 0.34 on the log scale (τ^2^ = 0.116), and the 95% prediction interval was 0.60–2.80; for all-cause mortality, τ = 0.32 (τ^2^ = 0.103) and the 95% prediction interval was 0.71–3.02. Both prediction intervals include 1.0 and are considerably wider than the corresponding confidence intervals, indicating that although the average association favored native-vessel PCI, the magnitude, and potentially the direction, of the association in an individual future setting is uncertain. The PROCTOR point estimates (RR 0.55 for MACE and 0.64 for mortality) lay just below the lower prediction limits, which suggests that differences between randomized and observational designs may contribute to the observed heterogeneity. With the Hartung–Knapp–Sidik–Jonkman adjustment, the confidence intervals remained above 1.0 for MACE (RR 1.30, 95% CI 1.03–1.63; *p* = 0.031) and all-cause mortality (RR 1.47, 95% CI 1.09–1.97; *p* = 0.014).

Risk-of-bias judgments are summarized in Section 3.6 and incorporated into the GRADE assessments (Table 3 and Table 4). The contributing studies varied in design, clinical presentation, anatomy, and follow-up, and the nonrandomized evidence was judged to have a serious overall risk of bias, principally because of confounding.

### 3.3. Revascularization Endpoints and Procedural Characteristics

Funnel plots for MACE and all-cause mortality are presented in Figure 1 and Figure 2, respectively. Egger’s test was applied where the number of studies permitted; these assessments were considered supportive evidence regarding possible small-study or dissemination effects rather than definitive evidence of absence of reporting bias.

**Figure 10:**
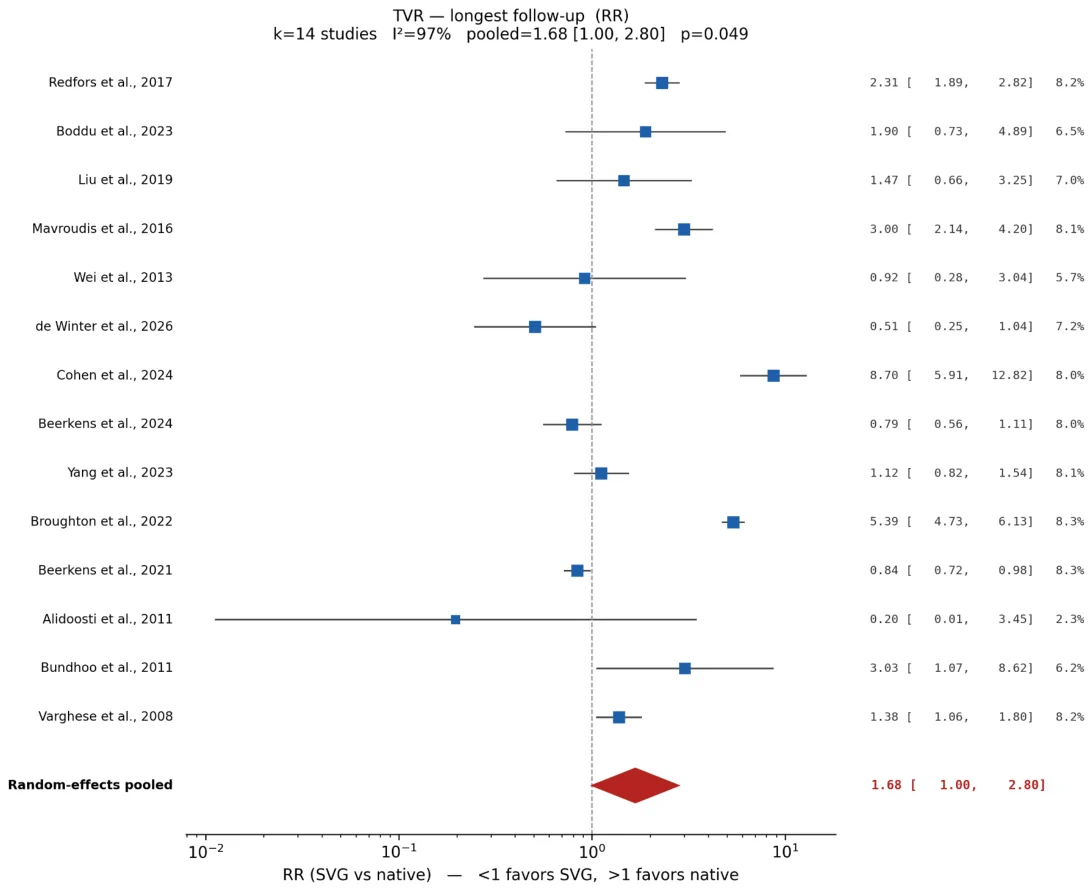
Forest plot of target vessel revascularization (TVR) comparing SVG PCI with native coronary artery PCI (random-effects meta-analysis) [7,22,24,25,29,30,31,32,33,34,38,39,40,41].

**Figure 11:**
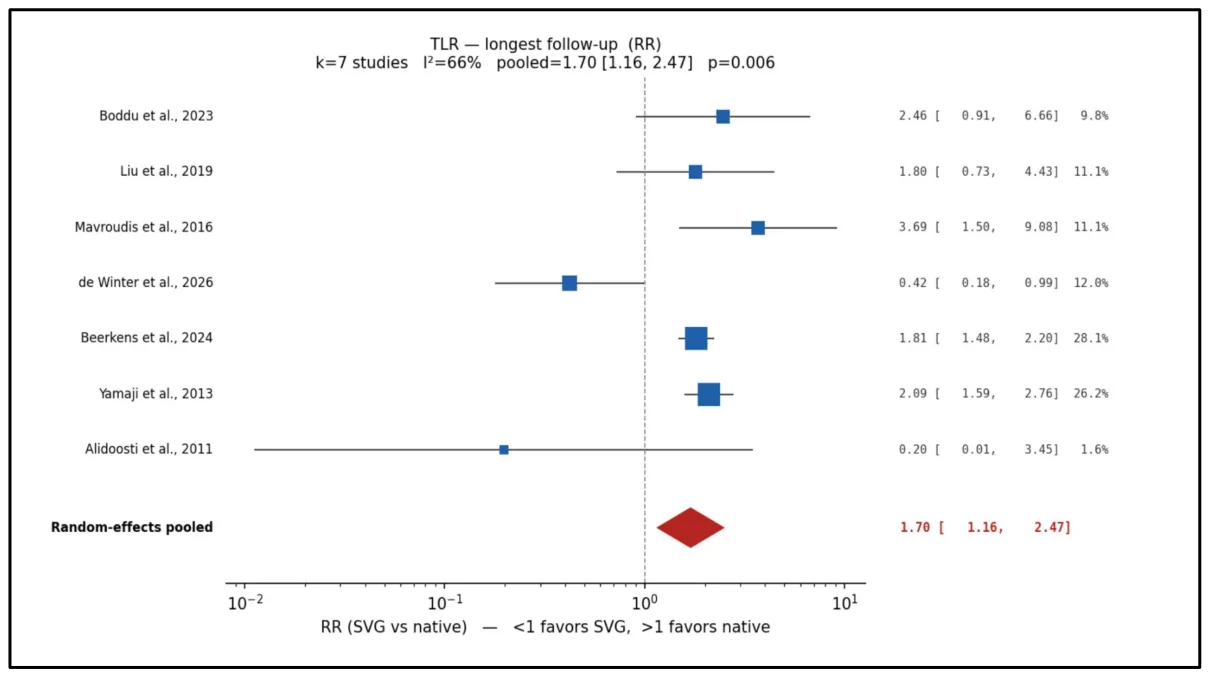
Forest plot of target lesion revascularization (TLR) comparing SVG PCI with native coronary artery PCI (random-effects meta-analysis) [7,25,27,29,30,33,38].

**Figure 12:**
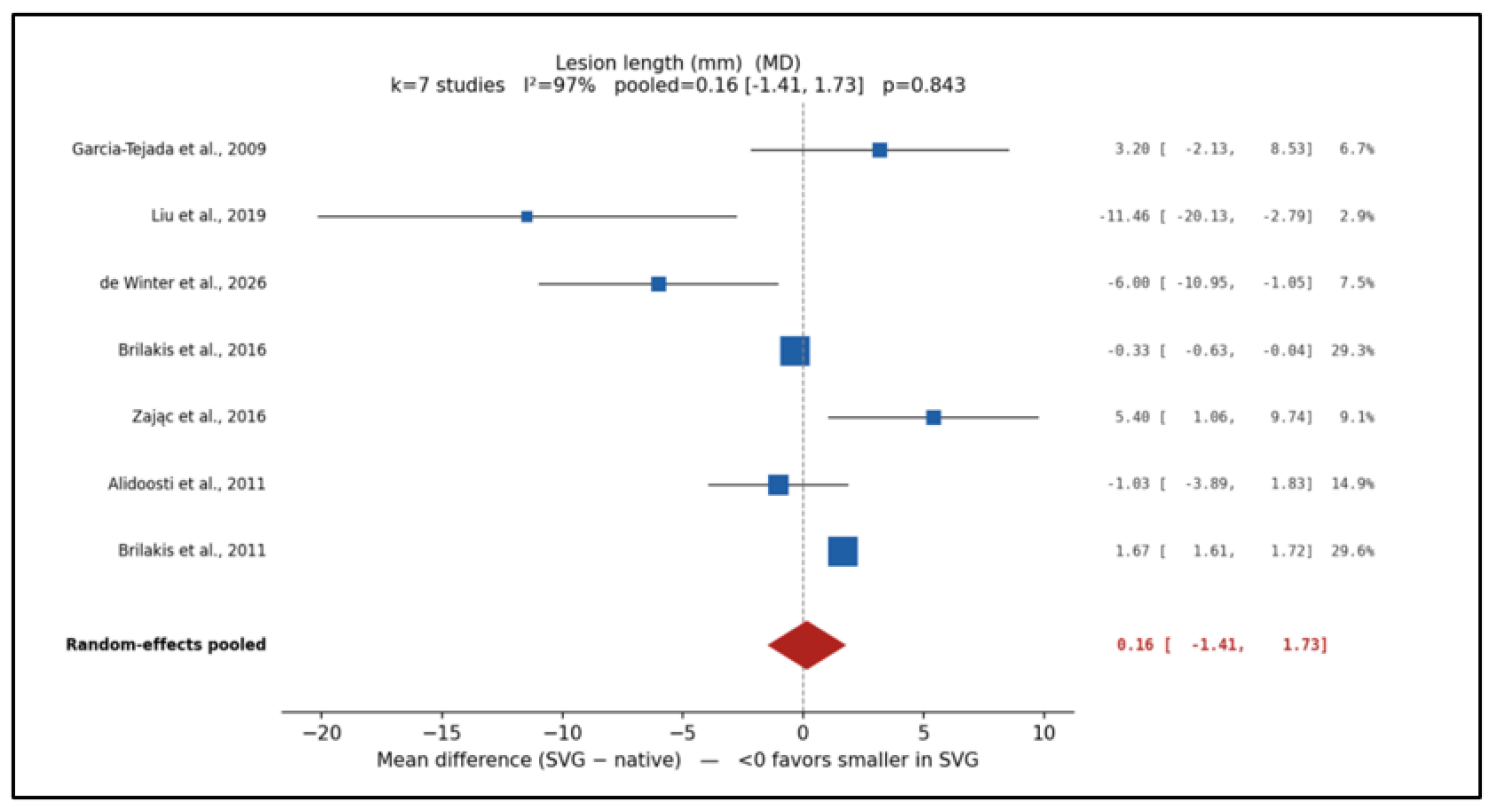
Forest plot of lesion length comparing SVG PCI with native coronary artery PCI (random-effects meta-analysis of mean differences) [5,7,23,25,28,30,35].

**Figure 13:**
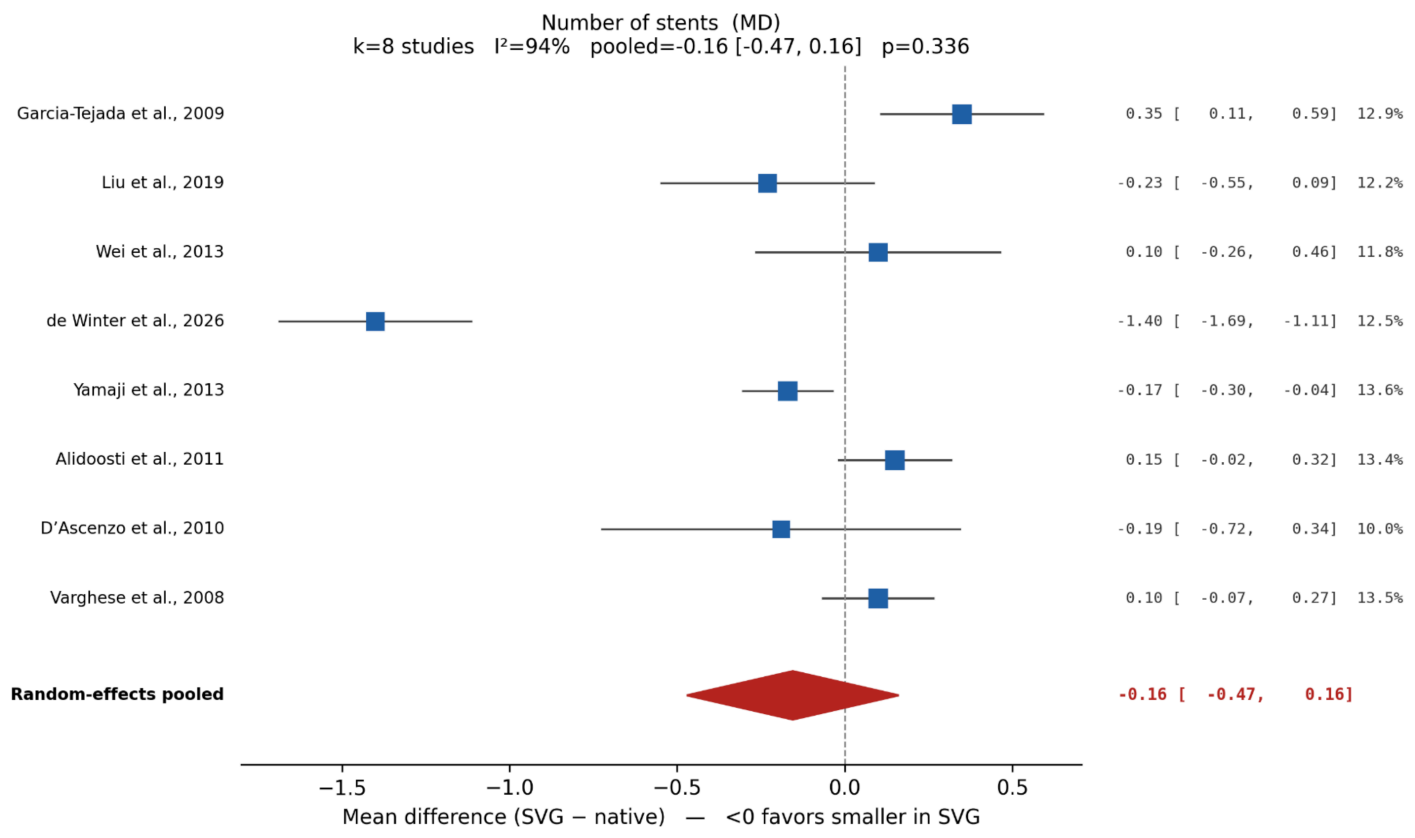
Forest plot of number of implanted stents comparing SVG PCI with native coronary artery PCI (random-effects meta-analysis of mean differences) [7,21,22,25,27,28,30,40].

**Figure 14:**
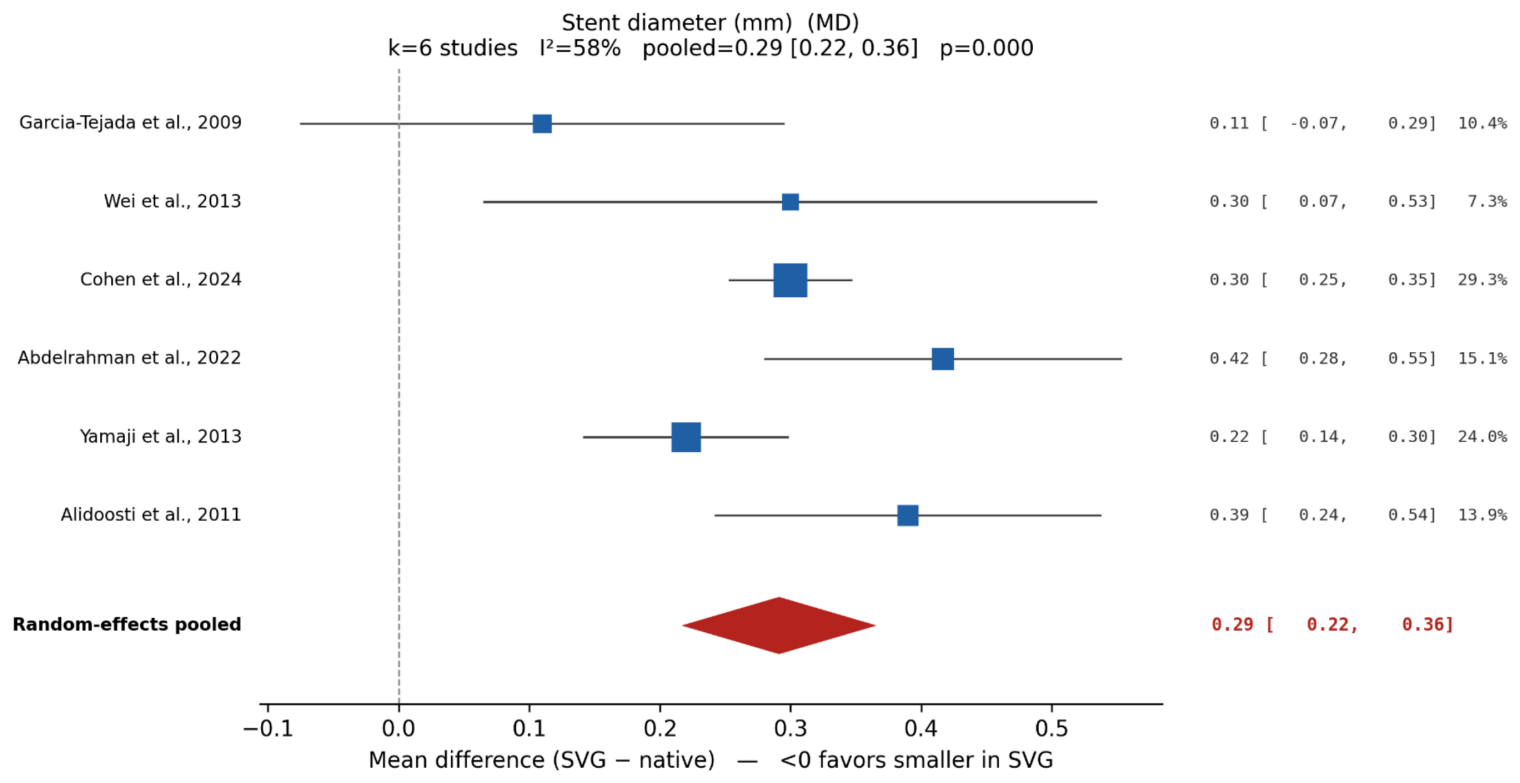
Forest plot of stent diameter comparing SVG PCI with native coronary artery PCI (random-effects meta-analysis of mean differences) [19,25,27,28,34,40].

**Figure 15:**
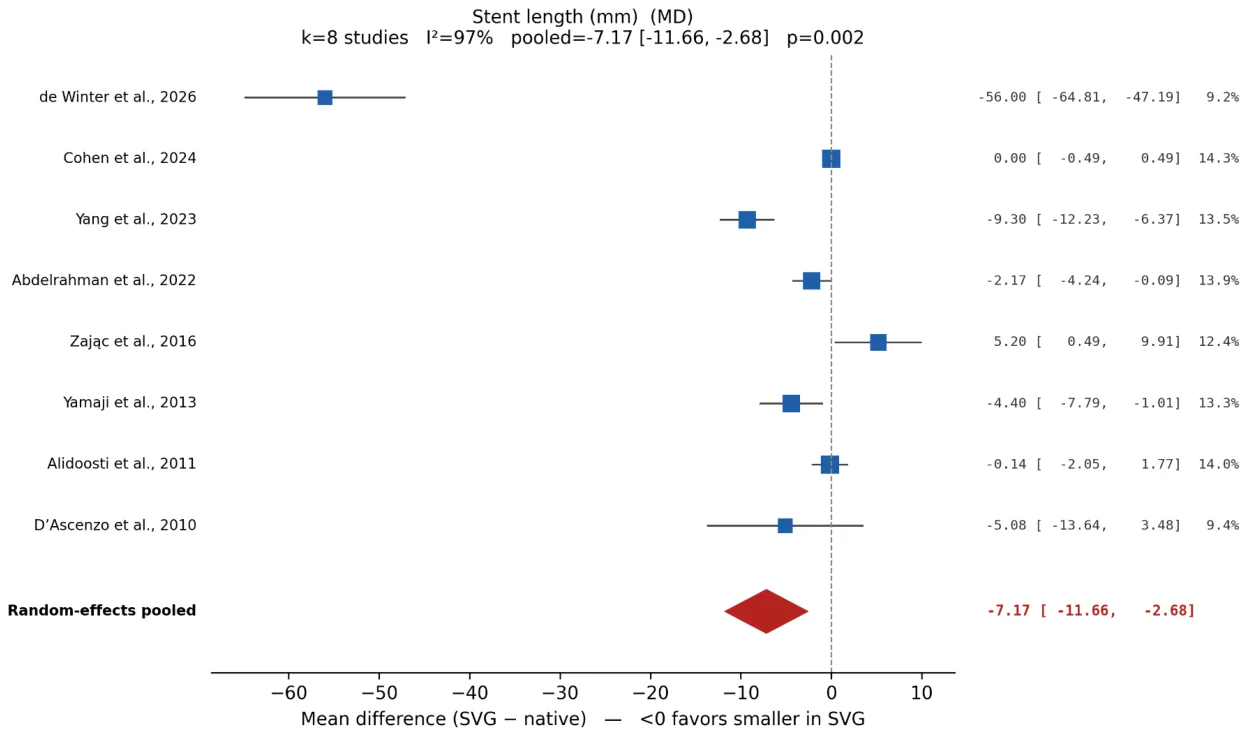
Forest plot of stent length comparing SVG PCI with native coronary artery PCI (random-effects meta-analysis of mean differences) [7,19,21,25,27,32,34,35].

TLR was significantly higher with SVG PCI (RR 1.70, 95% CI 1.16–2.47; *p* = 0.006; I^2^ = 66%; k = 7), and TVR showed a borderline significant increase (RR 1.68, 95% CI 1.00–2.80; *p* = 0.049; I^2^ = 97%; k = 14). SVG lesions treated required larger-diameter stents (MD +0.29 mm, 95% CI 0.22–0.36; *p* < 0.001; I^2^ = 58%; k = 6) but shorter total stent length (MD −7.17 mm, 95% CI −11.66 to −2.68; *p* = 0.002; I^2^ = 97%; k = 8). Lesion length (MD +0.16 mm, 95% CI −1.41 to 1.73; k = 7) and number of stents used (MD −0.16, 95% CI −0.47 to 0.16; k = 8) did not differ significantly between arms. Forest plots of revascularization and procedural outcomes are shown in Figure 10, Figure 11, Figure 12, Figure 13, Figure 14 and Figure 15.

**Figure 16:**
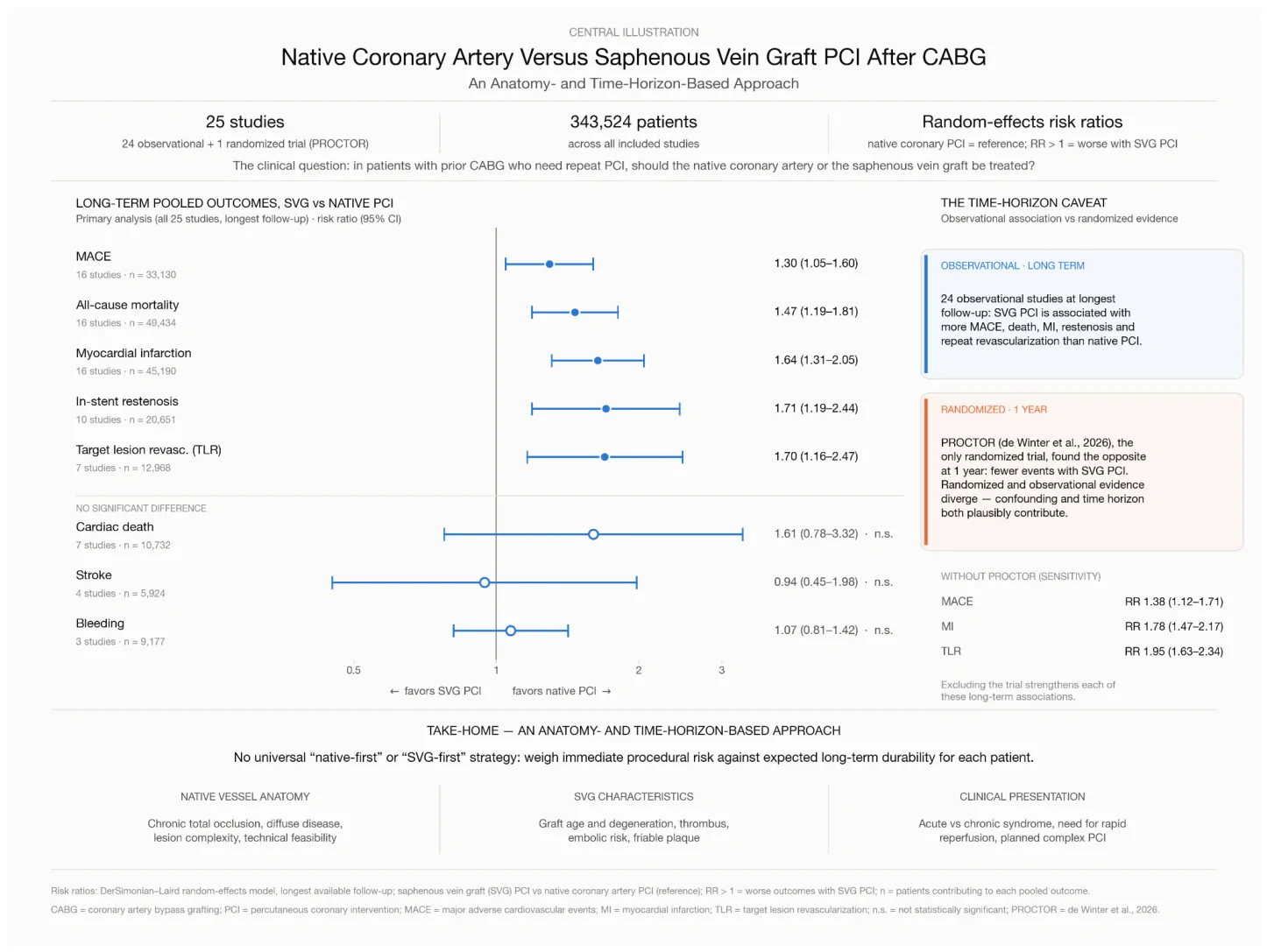
Central Illustration. Native coronary artery versus saphenous vein graft PCI after CABG: an anatomy- and time-horizon-based approach. Pooled risk ratios (DerSimonian–Laird random-effects model, longest available follow-up) are shown for SVG PCI versus native coronary artery PCI (reference); RR > 1 indicates worse outcomes with SVG PCI. All values correspond to the 25-study analysis reported in Section 3.2; the number of contributing studies and patients is shown beside each estimate (MACE, 16 studies, n = 33,130; all-cause mortality, 16 studies, n = 49,434; MI, 16 studies, n = 45,190; in-stent restenosis, 10 studies, n = 20,651; TLR, 7 studies, n = 12,968).

For the outcomes with the highest heterogeneity, the 95% prediction intervals were 0.21–13.14 for TVR (I^2^ = 97%) and −23.01 to 8.67 mm for stent length (I^2^ = 97%); the corresponding intervals for MI, ISR, TLR, and stent diameter were 0.83–3.25, 0.59–4.95, 0.61–4.72, and 0.09 to 0.49 mm, respectively. Where the prediction interval crosses the null (or zero for mean differences), the pooled estimate should be interpreted as an average across heterogeneous settings rather than a consistent effect. With the Hartung–Knapp–Sidik–Jonkman adjustment, the associations for MI (95% CI 1.26–2.13; *p* = 0.001) and ISR (95% CI 1.03–2.83; *p* = 0.041) remained statistically significant, whereas those for TLR (95% CI 0.90–3.20; *p* = 0.088), TVR (95% CI 0.95–2.95; *p* = 0.070), and stent length (95% CI −21.55 to 7.22 mm; *p* = 0.277) did not. The principal findings are summarized in the Central Illustration (Figure 16).

### 3.4. Sensitivity Analysis

Complete leave-one-out results for all pooled outcomes are reported in Supplementary Table S1. The findings for all-cause mortality (range of pooled RRs 1.39–1.54), MI (1.51–1.78), ISR (1.50–1.94), and stent diameter remained statistically significant after every single-study omission and were classified as robust. MACE (range 1.18–1.38) was classified as fragile because omission of Beerkens et al., 2024 [33] yielded RR 1.29 (95% CI 0.98–1.68; *p* = 0.070). TLR (range 1.52–1.95) was also fragile: omission of Beerkens et al., 2024 [33] (RR 1.56, 95% CI 0.83–2.95; *p* = 0.171) or Yamaji et al., 2013 [27] (RR 1.52, 95% CI 0.83–2.76; *p* = 0.172) removed statistical significance, whereas omission of de Winter et al., 2026 [7] strengthened the association (RR 1.95, 95% CI 1.63–2.34; I^2^ = 8%). For stent length, the pooled MD was −7.17 mm (95% CI −11.66 to −2.68) and the signed leave-one-out range was −9.19 to −2.14 mm; the point estimate therefore remained negative in every omission, but the result was classified as fragile according to the prespecified criterion (Section 2.3) because omission of de Winter et al., 2026 [7] yielded MD −2.14 mm (95% CI −4.67 to 0.40; *p* = 0.099). TVR, which was only marginally significant in the full model (*p* = 0.049), was also fragile. Cardiac death, stroke, bleeding, lesion length, and number of stents were not significant in the full model and remained non-significant on sensitivity testing.

Excluding the PROCTOR trial yielded modestly stronger associations for every outcome to which it contributed (Supplementary Table S2): MACE, RR 1.38 (95% CI 1.12–1.71; *p* = 0.003); all-cause mortality, RR 1.50 (95% CI 1.21–1.85); MI, RR 1.78 (95% CI 1.47–2.17), with I^2^ falling from 65% to 52%; TLR, RR 1.95 (95% CI 1.63–2.34), with I^2^ falling from 66% to 8%; and TVR, RR 1.84 (95% CI 1.08–3.12). The stent-length difference was attenuated and no longer statistically significant (MD −2.14 mm, 95% CI −4.67 to 0.40). PROCTOR did not contribute to the ISR, cardiac death, stroke, bleeding, or stent-diameter analyses, which were therefore unchanged.

### 3.5. Subgroup Analysis: DES-Predominant

Stent-type data were available for only 12 of the 25 total studies, of which 10 were DES-predominant, and 2 were BMS/mixed; 13 studies had no stent-type data available for classification. This distribution was insufficient for a meaningful between-group comparison.

In the DES-predominant subgroup alone, analyzed as risk ratios for consistency with all other analyses, effect directions were consistent with the overall pooled analysis for MI (RR 1.72, 95% CI 1.55–1.91; I^2^ = 0%; k = 6), MACE (RR 1.23, 95% CI 1.02–1.49; I^2^ = 22%; k = 6), TLR (RR 2.58, 95% CI 1.27–5.21; k = 2), and TVR (RR 2.37, 95% CI 1.21–4.64; k = 6). All-cause mortality (RR 1.42, 95% CI 0.90–2.23), ISR (RR 1.90, 95% CI 0.85–4.22), and cardiac death (RR 2.03, 95% CI 0.90–4.60) were directionally consistent but not statistically significant, likely reflecting reduced power.

### 3.6. Risk of Bias

Risk-of-bias judgments for each included study, by domain and overall, are presented in Supplementary Tables S5 and S6, summarized below, and incorporated into the GRADE ratings (Table 3 and Table 4). All 24 nonrandomized studies were judged at serious overall risk of bias, driven by confounding (Supplementary Table S5). The one-year PROCTOR MACE result was judged to raise some concerns overall. The randomization process, adherence to the intention-to-treat principle, and completeness of follow-up were judged at low risk; some concerns remained for outcome measurement and selection of the reported result because treatment was open label, clinically driven revascularization contributed to the composite endpoint, and the dated protocol, statistical analysis plan, and adjudication materials require confirmation for the one-year analysis. The all-cause mortality result was less susceptible to outcome-measurement bias but retained some concerns regarding selection of the reported result.

The nonrandomized clinical comparisons were judged to be at serious overall risk of bias, driven principally by confounding. Target-vessel selection depended on clinical acuity, native-vessel complexity and feasibility, graft age and morphology, thrombus burden, and operator judgment. These factors were not adequately controlled in the unadjusted event-count estimates used for the primary meta-analysis. Intervention classification was generally reliable when the treated vessel was captured prospectively in procedural records, whereas reporting of missing outcomes, crossover after unsuccessful PCI attempts, endpoint adjudication, and prespecified analyses was incomplete in several reports. Consequently, pooled observational risk ratios should be interpreted as associations rather than causal treatment effects.

Applying GRADE (Table 3 and Table 4), the certainty of the evidence from the primary pooled analyses was very low for all outcomes. All outcomes were rated down two levels for risk of bias because every contributing nonrandomized study had serious confounding. MACE, all-cause mortality, MI, ISR, TLR, TVR, and cardiac death were further rated down for inconsistency (I^2^ 65–97%, with prediction intervals that included no effect), and TVR, cardiac death, stroke, and bleeding for imprecision. When PROCTOR was excluded, heterogeneity fell for MI (I^2^ = 51%; RR 1.78, 95% CI 1.47–2.17) and TLR (I^2^ = 8%; RR 1.95, 95% CI 1.63–2.34) and their prediction intervals excluded no effect, so certainty for these two outcomes rose to low; it remained very low for all other outcomes (MACE: RR 1.38, 95% CI 1.12–1.71; all-cause mortality: RR 1.50, 95% CI 1.21–1.85). For the randomized evidence from PROCTOR alone, certainty was low for 1-year MACE and MI and very low for all-cause mortality, reflecting some concerns about risk of bias and imprecision after early termination at 220 of 584 planned patients.

## 4. Discussion

### 4.1. Principal Findings

In this updated meta-analysis of 25 studies comparing native coronary artery PCI with SVG PCI in patients with prior CABG, SVG PCI was associated with higher rates of MACE, all-cause mortality, MI, ISR, and TLR over long-term follow-up. The associations for mortality, MI, and ISR were robust in leave-one-out analyses, whereas those for MACE and TLR depended on individual large studies, and residual confounding and heterogeneity across the included studies cannot be excluded. The direction of association was also broadly consistent in the exploratory DES-predominant subgroup.

Overall, these findings are consistent with prior observational evidence suggesting more favorable long-term outcomes with native coronary artery PCI and provide context for interpreting the recently reported PROCTOR trial. The observational and randomized data are complementary rather than directly contradictory, reflecting differences in study design, patient selection, anatomical complexity, and follow-up duration. Given the predominantly observational nature of the available evidence, these findings should not be interpreted as establishing a causal advantage of native coronary artery PCI over SVG PCI.

### 4.2. Pathophysiological Basis for Inferior SVG PCI Outcomes

The adverse outcomes with SVG PCI are biologically plausible. After arterial implantation, SVGs undergo endothelial injury, neointimal hyperplasia, and progressive lipid/inflammatory accumulation, evolving into accelerated atherosclerosis and graft failure—reported in 39–61% of grafts beyond 10 years [1,3]. Unlike native coronary atherosclerosis, SVG plaques are diffuse, lipid-rich, and friable, making them prone to disruption during instrumentation; this releases thrombotic and atheromatous debris distally, increasing no-reflow risk and MI, particularly in acute settings [3]. This explains why angiographically successful SVG stenting does not guarantee effective myocardial reperfusion, and why our analysis showed higher MI risk with SVG PCI.

This biology likely also underlies the higher ISR and TLR rates observed: SVG lesions harbor diffuse disease beyond the treated segment, with graft degeneration continuing post-PCI. While DES reduces early restenosis versus BMS in some SVG populations, long-term durability is less consistent than in native arteries—ISAR-CABG showed early DES benefit that attenuated over time, DELAYED RRISC found loss of early revascularization benefit with unexpected late mortality increase with sirolimus-eluting stents, and DIVA found no significant DES–BMS difference in target-vessel failure [42,43,44]. Together, these findings suggest vein graft biology itself may limit the durability of stent-based therapy regardless of stent generation.

### 4.3. Reconciling the Meta-Analysis with the PROCTOR Trial

The principal apparent difference between the present meta-analysis and the PROCTOR trial is the direction of the observed 1-year versus longer-term associations. Our pooled analysis found higher long-term MACE, mortality, MI, ISR, and TLR with SVG PCI, whereas PROCTOR demonstrated lower 1-year MACE with SVG PCI, primarily because of fewer PCI-related MI events and clinically driven target coronary territory revascularizations [7].

Several factors may explain these complementary findings. First, PROCTOR’s Heart Team-based randomization—requiring both targets to be deemed appropriate—reduced selection bias but produced a population that may not reflect routine post-CABG PCI practice [6,7]. Second, its native-vessel arm carried a high burden of complex anatomy, including CTOs, which are more technically demanding after CABG with lower procedural success [45]; the excess periprocedural MI seen with native PCI may thus reflect procedural complexity rather than a true disadvantage of the approach itself. Third, PROCTOR primarily reported 1-year outcomes, whereas our meta-analysis used each study’s longest available follow-up.

This distinction is particularly important for ISR and other late events, which may emerge beyond the first year, and makes direct comparison of 1-year randomized outcomes with longer-term pooled observational associations inappropriate. Fourth, competitive flow may modulate outcomes, as a patent graft can alter native hemodynamics and stent patency while also serving as a perfusion safety net during difficult native PCI [46]. Finally, PROCTOR was stopped early (220 of a planned 584 patients), increasing uncertainty around secondary and subgroup estimates; longer follow-up remains important, as SVG’s biological disadvantages may only become apparent with time [6,7]. Together, these factors suggest the optimal target cannot be decided by location alone, but must weigh graft status, native anatomy, lesion complexity, and procedural risk.

Our sensitivity analysis quantifies this divergence. Excluding PROCTOR, the pooled MACE estimate shifted from RR 1.30 to 1.38, and the mortality estimate from 1.47 to 1.50, and heterogeneity among the remaining observational studies was essentially unchanged (I^2^ = 85% for MACE and 91% for mortality). The randomized trial therefore did not account for the overall heterogeneity in these outcomes, but it was the main reason the pooled estimates were attenuated toward the null; for MI and TLR, by contrast, excluding PROCTOR also reduced heterogeneity substantially (I^2^ from 65% to 52% and from 66% to 8%, respectively). A single trial of 220 patients with 1-year follow-up and a body of observational cohorts with longer follow-up address different questions: the nonrandomized-only estimates are the best summary of long-term observational associations, whereas PROCTOR provides the best available estimate of the short-term causal effect in Heart Team-selected patients in whom both targets are feasible.

### 4.4. Clinical Implications

Taken together, the available evidence supports a nuanced rather than absolute native-vessel-first strategy. The observational literature and our updated meta-analysis favor native coronary artery PCI when technically feasible, consistent with current ACC/AHA/SCAI guideline recommendations [4]. Similarly, PROCTOR demonstrates that native-vessel PCI is not universally superior, particularly when the native target is highly complex, and the SVG has favorable anatomy.

Target-vessel selection should therefore be individualized according to native-vessel lesion complexity, CTO characteristics, graft age and morphology, presence of thrombus or diffuse graft degeneration, myocardial territory at risk, prior stent anatomy, and the operator’s anticipated procedural risk. In patients with complex native CTOs and a relatively favorable SVG target, SVG PCI may provide a lower-risk short-term procedure [47,48]. Conversely, severely degenerated or occluded SVGs may carry substantial embolic and restenotic risk, making native-vessel PCI preferable when technically achievable [49].

In ACS/STEMI, SVG PCI outcomes are comparable to native-vessel PCI, so emergent SVG PCI is reasonable when the native vessel cannot be accessed, though the slow/no-reflow risk is substantially higher (29% vs. ~15% in stable cases) [50]. In stable CCS, registries show worse long-term outcomes (death, MI, TVR at 2–3 years) with SVG PCI, favoring a native-first strategy when there is time to plan, including CTO referral [4].

Presentation dictates urgency, not target vessel choice. In ACS with a degenerated culprit SVG, acute SVG PCI for reperfusion followed by staged native PCI may be considered if native vessel PCI is technically difficult; in CCS, native-first is preferred since there is time to plan. Thus, a Heart Team-based, anatomy-driven approach that explicitly considers both immediate procedural risk and expected long-term durability is likely more appropriate than a rigid strategy favoring either target in all patients.

### 4.5. Special Consideration: PCI at the Graft-Native Vessel Anastomosis

Our analysis compares target vessels, whereas lesions at or immediately adjacent to the distal graft-to-native anastomosis do not fit neatly into either category. Distal anastomotic lesions are among the most common causes of both venous and arterial graft failure [51]. Many arise from technical factors at the time of surgery, such as suture-line tension, anastomotic angulation, size mismatch between graft and recipient vessel, or injury to the native vessel during grafting, superimposed on neointimal hyperplasia at the suture line [51,52]. Because redo CABG carries higher risks of mortality and myocardial infarction, percutaneous treatment is often preferred, although the optimal technique has not been established [51,53].

These lesions can be approached through the graft, retrogradely from the native vessel, or with a combined strategy, and the angiographic result is not always optimal [52]. Embolic protection devices that require a distal landing zone generally cannot be used in distal anastomotic lesions unless deployed in the native vessel [2]. The choice depends on graft type (SVG versus internal mammary artery), graft tortuosity and angulation, the interval since surgery, and whether the native vessel can be wired safely. It also interacts with the native-versus-graft question addressed in this review: treating an anastomotic lesion via the native vessel avoids manipulation of a diseased graft and its embolic risk but requires wiring across a freshly sutured and potentially fragile segment.

Perforation is the principal complication that makes this decision difficult. The suture line and the adjacent recipient vessel may be thin-walled, angulated, or fibrotic, and stiff-wire manipulation, aggressive predilatation, and oversized balloons or stents can each cause vessel rupture. Prior CABG is also associated with a higher risk of perforation during CTO PCI [54,55]. Two features of the post-CABG setting affect how perforation should be judged. First, pericardial adhesions may contain bleeding and reduce the risk of classic tamponade, but loculated effusions can still compress cardiac chambers and cause hemodynamic collapse, so early recognition and sealing are important [54]. Second, perforation involving a bypass graft is uncommon and can generally be sealed with covered stents [56,57]; however, covered stents are less deliverable in tortuous anastomotic segments and may occlude side branches or jump-graft outflow.

These considerations favor a cautious technical strategy: careful angiographic assessment of the anastomosis and native run-off; intravascular imaging when feasible; soft-tip or polymer-jacketed wires; undersized initial balloon dilatation, with low-pressure plain balloon angioplasty as a lower-risk alternative to stenting in selected cases [52]; immediate availability of covered stents and embolization equipment; and a low threshold for echocardiographic reassessment and pericardiocentesis if the patient deteriorates. Evidence for anastomotic PCI is limited to small series and case reports, without randomized data or comparative studies of access route or stent type [51,52,53]. Decisions should therefore be made by the Heart Team, weighing technical difficulty, perforation risk, and the alternatives of redo surgery or medical therapy, rather than by a fixed rule. Importantly, none of the studies in our meta-analysis stratified outcomes by anastomotic versus non-anastomotic lesion location, so our pooled estimates cannot be extended to anastomotic lesions, which represents an important evidence gap.

### 4.6. Limitations

Several limitations should be considered. Most included studies were observational, creating a serious risk of selection bias, confounding by indication, and residual confounding. In clinical practice, selection of native vessel versus SVG PCI is influenced by graft condition, native coronary anatomy, lesion complexity, procedural feasibility, myocardium at risk, comorbidity burden, and operator expertise. These factors also affect mortality, myocardial infarction, and repeat revascularization. Consequently, pooled unadjusted estimates should be interpreted as associations rather than causal effects of revascularization strategy.

Substantial heterogeneity was also present for several outcomes, likely reflecting differences in endpoint definitions, lesion and graft characteristics, follow-up duration, and evolving PCI techniques. The absence of individual patient-level data prevented adjustment for important lesion- and procedure-level factors, including CTO complexity, graft age and morphology, thrombus burden, embolic protection, intravascular imaging, and operator experience. Incomplete reporting of stent type further limited interpretation of the DES-predominant subgroup, while variable follow-up precluded reliable duration-based analyses.

The robustness of the pooled estimates in the presence of this heterogeneity warrants explicit consideration. Heterogeneity was very high for TVR (I^2^ = 97%) and stent length (I^2^ = 97%) and also substantial for all-cause mortality (I^2^ = 90%) and MACE (I^2^ = 86%). First, with such values, the random-effects summary represents an average of effects that differ considerably across studies rather than a single common effect, and it should not be assumed to apply to any individual clinical setting. Second, the 95% prediction intervals for MACE (0.60–2.80) and all-cause mortality (0.71–3.02) included 1.0, so the true association in a new population could be null or even favor SVG PCI, as suggested by PROCTOR; the statistically significant pooled estimates therefore indicate a consistent average direction, not a uniform effect. Third, the DerSimonian–Laird estimator with Wald-type confidence intervals can understate uncertainty when between-study variance is large; although the Hartung–Knapp–Sidik–Jonkman-adjusted intervals for MACE, mortality, MI, and ISR remained above 1.0, the TLR, TVR, and stent-length findings lost statistical significance with this adjustment and were also fragile in leave-one-out analysis; these findings, including the borderline TVR result (lower confidence limit 1.00; *p* = 0.049; I^2^ = 97%), should be regarded as inconclusive. Fourth, I^2^ may be inflated when several large studies with very precise estimates are pooled, and part of the heterogeneity for procedural outcomes such as stent length likely reflects differences in measurement and operator practice rather than true biological variation. Finally, because meta-regression was not feasible, we could not formally quantify how study design (randomized vs. observational), clinical presentation, stent type, or follow-up duration contributed to heterogeneity. Taken together, these findings are best interpreted as consistent in direction but imprecise in magnitude, and as hypothesis-generating rather than as definitive estimates of effect.

Importantly, statistical significance, low heterogeneity, and leave-one-out stability do not eliminate structural confounding shared across observational cohorts. The randomized PROCTOR trial addresses this limitation more directly but enrolled a selected population in whom both PCI strategies were considered feasible and was terminated early with 1-year follow-up. Randomized and observational evidence should therefore be interpreted separately, recognizing their differing populations, outcome definitions, and follow-up periods.

Additional review-process limitations include reliance on published aggregate data, incomplete reporting of study-level covariates and stent type, and the inability to perform a formal meta-regression or individual-patient-data analysis. Using GRADE, the certainty of evidence was low to very low for all outcomes in both the nonrandomized and randomized bodies of evidence; the findings should therefore be regarded as associations of uncertain magnitude, further research is very likely to change the estimates, and the true effects may be substantially different from those reported here, including for outcomes in which the pooled association was statistically significant. In addition, the included studies did not report outcomes by lesion location within the graft-native vessel complex, so we could not evaluate anastomotic lesions separately (see Section 4.5).

## 5. Future Directions

Future randomized trials should evaluate native-vessel and SVG PCI across a broader range of post-CABG anatomy and should include sufficiently long follow-up to capture late graft degeneration and repeat revascularization. Contemporary studies should incorporate intravascular imaging, modern CTO techniques, standardized definitions of periprocedural MI, and systematic assessment of embolic protection strategies.

Importantly, trials should distinguish between patients with severely degenerated SVGs and those with relatively favorable graft anatomy, as these groups may have fundamentally different risk-benefit profiles.

Prospective registries and trials should also report lesion location (ostial, body, or anastomotic), access route, and the occurrence and management of perforation, to inform target selection for anastomotic lesions after CABG.

Until such evidence is available, the preponderance of long-term observational and meta-analytic evidence supports native coronary artery PCI when technically feasible, while the PROCTOR trial appropriately highlights the need for individualized target selection in patients with complex native coronary anatomy and favorable SVG targets.

## 6. Conclusions

The current meta-analysis of mostly observational studies suggests that in patients with CABG undergoing PCI, SVG PCI was associated with worse long-term outcomes compared to native artery PCI (certainty evidence low to very low), including higher rates of MACE, all-cause mortality, MI, ISR, and TLR. Associations for mortality, MI, and ISR were robust to leave-one-out analyses, whereas those for MACE and TLR were sensitive to the omission of single studies; all remained directionally consistent in DES-predominant studies, whereas the randomized PROCTOR trial reported lower 1-year MACE with SVG PCI. Taken together, these findings should be interpreted as complementary evidence across different clinical and temporal contexts rather than as evidence that one strategy is uniformly superior. Further adequately powered randomized trials with extended follow-up are needed to define the optimal revascularization strategy across the spectrum of post-CABG anatomy. In practical terms, native-vessel PCI is preferred when technically feasible, particularly in stable disease with a degenerated graft, whereas SVG PCI remains reasonable when the native target is a complex CTO and the graft anatomy is favorable, or in acute coronary syndrome when the native vessel cannot be treated promptly. Lesions at the graft-native anastomosis represent a distinct, technically demanding scenario with a substantial risk of perforation, for which comparative data are lacking, and Heart Team decision-making is essential.

## Figures and Tables

**Table 1:** MACE—study-level risk ratios (random-effects meta-analysis). DerSimonian–Laird random-effects model; outcome assessed at each study’s longest reported follow-up. RR > 1 indicates a higher MACE rate with SVG relative to native-vessel PCI. I^2^ = 86%; *p* = 0.017.

Study	Design	RR [95% CI] (SVG vs. Native)	Weight
Redfors et al., 2017 [39]	Prospective multicenter Cohort Study	3.06 [2.44, 3.83]	9.0%
Boddu et al., 2023 [38]	Retrospective Cohort Study	1.79 [1.20, 2.68]	7.4%
Liu et al., 2019 [30]	Retrospective Cohort Study	1.23 [0.66, 2.30]	5.4%
Tejada et al., 2008 [36]	Retrospective single center (Madrid)	0.88 [0.35, 2.23]	3.5%
Wei et al., 2013 [40]	Retrospective Cohort Study	1.47 [0.72, 2.99]	4.7%
de Winter et al., 2026 [7]	Randomized Control Trial	0.55 [0.34, 0.87]	6.8%
Cohen et al., 2024 [34]	Multicenter retrospective cohort study	0.94 [0.69, 1.29]	8.3%
Beerkens et al., 2024 [33]	Retrospective nationwide registry (Netherlands Heart Registration)	1.38 [1.26, 1.51]	9.9%
Yang et al., 2023 [32]	Retrospective, single center (Beijing)	1.29 [1.06, 1.57]	9.2%
Liu et al., 2023 [37]	Prospective cohort, single-center (China)	1.26 [0.85, 1.87]	7.5%
Beerkens et al., 2021 [31]	Retrospective single center study	0.96 [0.86, 1.08]	9.8%
Alidoosti et al., 2011 [25]	Observational cohort, single-center (Tehran)	0.97 [0.27, 3.54]	2.1%
Bundhoo et al., 2011 [24]	Retrospective, multicenter (UK)	2.43 [1.11, 5.34]	4.2%
D’Ascenzo et al., 2010 [21]	Observational cohort (Italy)	1.00 [0.57, 1.76]	5.9%
Eid-Lidt et al., 2009 [20]	Retrospective Cohort Study	1.50 [0.86, 2.63]	5.9%
Varghese et al., 2008 [22]	Retrospective single-center (Dallas VA)	3.92 [0.16, 95.21]	0.4%
Random-effects pooled (k = 16)	—	1.30 [1.05, 1.60]	100%

CI = confidence interval; MACE = major adverse cardiac events; RR = risk ratio; SVG = saphenous vein graft.

**Table 2:** All-cause mortality—study-level risk ratios (random-effects meta-analysis). DerSimonian–Laird random-effects model; outcome assessed at each study’s longest reported follow-up. RR > 1 indicates higher all-cause mortality with SVG relative to native-vessel PCI. I^2^ = 90%; *p* < 0.001.

Study	Design	RR [95% CI] (SVG vs. Native)	Weight
Redfors et al., 2017 [39]	Prospective multicenter cohort study	2.47 [1.77, 3.44]	8.7%
Boddu et al., 2023 [38]	Retrospective Cohort Study	1.97 [0.84, 4.64]	3.9%
Garcia-Tejada et al., 2009 [28]	Retrospective Cohort Study	7.36 [1.49, 36.30]	1.5%
Mavroudis et al., 2016 [29]	Retrospective single center (Madrid)	1.42 [0.84, 2.41]	6.5%
Wei et al., 2013 [40]	Retrospective Cohort Study	14.67 [1.70, 126.40]	0.9%
de Winter et al., 2026 [7]	Randomized Control Trial	0.64 [0.19, 2.22]	2.3%
Cohen et al., 2024 [34]	Multicenter retrospective cohort study	0.73 [0.65, 0.81]	10.8%
Beerkens et al., 2024 [33]	Retrospective nationwide registry (Netherlands Heart Registration)	1.48 [1.29, 1.70]	10.6%
Yang et al., 2023 [32]	Retrospective, single center (Beijing)	1.19 [0.77, 1.85]	7.5%
Liu et al., 2023 [37]	Prospective cohort, single-center (China)	7.35 [2.56, 21.13]	2.9%
Broughton et al., 2022 [41]	Retrospective single center study	1.23 [1.11, 1.36]	10.9%
Beerkens et al., 2021 [31]	Retrospective single center study	1.02 [0.83, 1.25]	10.1%
Brilakis et al., 2016 [5]	Retrospective multicenter registry (VA CART, USA)	1.43 [1.33, 1.54]	11.0%
Yamaji et al., 2013 [27]	Observational cohort (Japan)	1.57 [1.10, 2.23]	8.5%
D’Ascenzo et al., 2010 [21]	Observational cohort (Italy)	2.00 [0.84, 4.77]	3.8%
Varghese et al., 2008 [22]	Retrospective single-center (Dallas VA)	3.92 [0.16, 95.21]	0.4%
Random-effects pooled (k = 16)	—	1.47 [1.19, 1.81]	100%

CI = confidence interval; RR = risk ratio; SVG = saphenous vein graft.

**Table 3:** GRADE summary of findings—primary pooled analyses of SVG PCI versus native-vessel PCI. Random-effects (DerSimonian–Laird) pooling at each study’s longest reported follow-up, based on the 25 included studies, including PROCTOR. RR > 1 indicates higher risk with SVG PCI. Absolute effect: median native-arm risk → risk with SVG PCI (95% CI), per 1000 patients. Body rated as nonrandomized evidence; starting certainty high (ROBINS-I); ●○○○ very low.

Outcome	Studies (Participants)	RR (95% CI)	95% PI	I^2^	Absolute Effect Per 1000	Certainty (GRADE)	Reasons for Rating	Certainty Without PROCTOR *
MACE	16 (33,130)	1.30 (1.05–1.60)	0.60–2.80	86%	167 → 217 (175–268) +49 per 1000	●○○○ Very low	RoB −2; inconsistency −1 (I^2^ = 86%; PI spans 1); significance lost in 1 leave-one-out omission	Very low
All-cause mortality	16 (49,434)	1.47 (1.19–1.81)	0.71–3.02	90%	74 → 109 (89–134) +35 per 1000	●○○○ Very low	RoB −2; inconsistency −1 (I^2^ = 90%; PI spans 1)	Very low
Myocardial infarction	16 (45,190)	1.64 (1.31–2.05)	0.83–3.25	65%	42 → 68 (55–85) +27 per 1000	●○○○ Very low	RoB −2; inconsistency −1 (I^2^ = 65%; PI spans 1)	Low
In-stent restenosis	10 (20,651)	1.71 (1.19–2.44)	0.59–4.95	73%	34 → 58 (40–83) +24 per 1000	●○○○ Very low	RoB −2; inconsistency −1 (I^2^ = 73%; PI spans 1)	Very low
Cardiac death	7 (10,506)	1.61 (0.78–3.32)	0.19–13.71	77%	27 → 43 (21–89) +16 per 1000	●○○○ Very low	RoB −2; inconsistency −1 (I^2^ = 77%); imprecision −1 (CI includes no effect and appreciable harm)	Very low
Stroke	4 (5840)	0.94 (0.45–1.98)	0.06–14.90	48%	33 → 31 (15–65) −2 per 1000	●○○○ Very low	RoB −2; imprecision −2 (4 studies; CI 0.45–1.98)	Very low
Bleeding	3 (9177)	1.07 (0.81–1.42)	0.18–6.55	0%	90 → 97 (73–128) +7 per 1000	●○○○ Very low	RoB −2; imprecision −1 (3 studies; wide CI)	Very low
Target lesion revascularization	7 (12,968)	1.70 (1.16–2.47)	0.61–4.72	66%	72 → 123 (84–179) +50 per 1000	●○○○ Very low	RoB −2; inconsistency −1 (I^2^ = 66%; PI spans 1); not robust to leave-one-out	Low
Target vessel revascularization	14 (36,874)	1.68 (1.00–2.80)	0.21–13.14	97%	97 → 162 (97–271) +65 per 1000	●○○○ Very low	RoB −2; inconsistency −1 (I^2^ = 97%; PI 0.21–13.1); imprecision −1 (lower CI limit 1.00)	Very low

* Pooling the 15 nonrandomized studies without PROCTOR: MI RR 1.78 (1.47–2.17), I^2^ = 51%, PI 1.06–3.01; TLR RR 1.95 (1.63–2.34), I^2^ = 8%, PI 1.42–2.70 (certainty low for both); MACE RR 1.38 (1.12–1.71) and mortality RR 1.50 (1.21–1.85) remained very low. CI = confidence interval; PI = prediction interval; RoB = risk of bias (−2 = rated down two levels for serious confounding in nonrandomized studies); RR = risk ratio; SVG = saphenous vein graft. GRADE certainty: ●●●● high; ●●●○ moderate; ●●○○ low; ●○○○ very low.

**Table 4:** GRADE summary of findings—randomized evidence (PROCTOR). RR < 1 indicates lower risk with SVG PCI. Absolute effect: native-arm risk in the trial → risk with SVG PCI (95% CI), per 1000 patients. Starting certainty: high.

Outcome	Studies (Participants)	RR (95% CI)	Absolute Effect Per 1000	Certainty (GRADE)	Reasons for Rating
MACE (1 year)	1 RCT (220)	0.55 (0.34–0.87)	343 → 188 (118–299) −155 per 1000	●●○○ Low	RoB −1 (some concerns: open-label; composite includes clinically driven revascularization); imprecision −1 (terminated early at 220 of 584 planned; optimal information size ≈ 890)
All-cause mortality (1 year)	1 RCT (220)	0.64 (0.19–2.22)	56 → 36 (10–123) −20 per 1000	●○○○ Very low	RoB −1 (some concerns: selection of reported result); imprecision −2 (10 deaths; CI compatible with large benefit and large harm)
Myocardial infarction (1 year)	1 RCT (220)	0.52 (0.28–0.97)	222 → 116 (62–216) −106 per 1000	●●○○ Low	RoB −1 (open label; endpoint dominated by periprocedural MI, definition-dependent); imprecision −1 (early termination; interval close to 1)

CI = confidence interval; RCT = randomized controlled trial; RoB = risk of bias; RR = risk ratio; SVG = saphenous vein graft. GRADE certainty: ●●●● high; ●●●○ moderate; ●●○○ low; ●○○○ very low.

## Data Availability

All data analyzed in this study were obtained from previously published studies. Data supporting the findings of this meta-analysis are available within the article and its Supplementary Materials. Additional extracted data are available from the corresponding author upon reasonable request.

## Supplementary Materials

The following supporting information can be downloaded at: https://www.mdpi.com/article/10.3390/jcm15218672/s1, Supplementary Methods S1: Search strategy; Table S1: Complete leave-one-out sensitivity analyses for all pooled outcomes; Table S2: Pooled estimates with and without the randomized PROCTOR trial; Table S3: 95% prediction intervals and Hartung–Knapp–Sidik–Jonkman sensitivity confidence intervals for all pooled outcomes; Table S4: Exploratory drug-eluting stent–predominant subgroup analysis; Table S5: ROBINS-I risk-of-bias assessment of the 24 nonrandomized studies, by domain and overall, with the basis for each judgment; Table S6: RoB 2 risk-of-bias assessment of the randomized PROCTOR trial, by domain and outcome; PRISMA 2020 checklist. References [5,7,19,20,21,22,23,24,25,26,27,28,29,30,31,32,33,34,35,36,37,38,39,40,41] are cited in the Supplementary Materials.
