# Supplementary material for: Native Coronary Artery Versus Saphenous Vein Graft Percutaneous Coronary Intervention in Patients with Prior Coronary Artery Bypass Grafting: An Updated Systematic Review and Meta-Analysis
Source: J Clin Med. 2026 Nov 6;15(21):8672. doi: 10.3390/jcm15218672 (PMC13764333; doi:10.3390/jcm15218672)
Supplement: Supplementary file 1 [file jcm-15-08672-s001.zip › jcm-4579736-supplementary.pdf]

SUPPLEMENTARY DATA

Native Coronary Artery Versus Saphenous Vein Graft Percutaneous Coronary Intervention in Patients With Prior Coronary Artery Bypass Grafting: An Updated Systematic Review and Meta-Analysis

Supplementary Methods S1. Search Strategy

PubMed

```
(
("Coronary Artery Bypass"[Mesh]
OR "Coronary Artery Bypass Grafting"[Mesh]
OR CABG[tiab]
OR "coronary artery bypass"[tiab]
OR "coronary bypass"[tiab]
OR "prior CABG"[tiab]
OR "previous CABG"[tiab]
OR "post-CABG"[tiab]
OR "status post CABG"[tiab])
AND
("Percutaneous Coronary Intervention"[Mesh]
OR PCI[tiab]
OR "percutaneous coronary intervention"[tiab]
OR angioplasty[tiab]
OR stent*[tiab])
AND
("native coronary artery"[tiab]
OR "native coronary"[tiab]
OR "native vessel"[tiab]
OR "native artery"[tiab]
OR "native lesion"[tiab])
AND
("saphenous vein graft"[tiab]
OR SVG[tiab]
OR "vein graft"[tiab]
OR "bypass graft"[tiab]
OR "graft vessel"[tiab]
OR "graft PCI"[tiab]
OR "SVG PCI"[tiab])
)
NOT (review[pt] OR editorial[pt] OR letter[pt] OR comment[pt])
NOT (animals[mh] NOT humans[mh])
```

Embase

```
(
'exp coronary artery bypass graft'/exp
OR CABG:ti,ab
OR 'coronary artery bypass':ti,ab
OR 'previous CABG':ti,ab
OR 'prior CABG':ti,ab
OR 'post-CABG':ti,ab
)
AND
(
'exp percutaneous coronary intervention'/exp
OR PCI:ti,ab
OR 'percutaneous coronary intervention':ti,ab
)
AND
(
'native coronary artery':ti,ab
OR 'native coronary':ti,ab
OR 'native vessel':ti,ab
)
AND
(
'exp saphenous vein graft'/exp
OR 'saphenous vein graft':ti,ab
OR SVG:ti,ab
OR 'vein graft':ti,ab
OR 'graft PCI':ti,ab
```

```
OR 'SVG PCI':ti,ab
)
NOT ('conference abstract'/it OR 'conference review'/it OR editorial:it OR letter:it)
NOT ('animal'/exp NOT 'human'/exp)
```

Cochrane

```
(Coronary Artery Bypass
OR CABG
OR coronary artery bypass
OR previous CABG OR prior CABG OR post-CABG)
AND
(Percutaneous Coronary Intervention
OR PCI
OR percutaneous coronary intervention)
AND
(native coronary artery
OR native coronary
OR native vessel)
AND
(Saphenous Vein
OR saphenous vein graft OR SVG
OR vein graft
OR graft PCI
OR SVG PCI)
```

Supplementary Tables

General statistical note: All analyses used a DerSimonian-Laird random-effects model on the corrected 25-study included set (Waldo et al., 2013; Meliga et al., 2007; and Ernst et al., 1986 excluded). The reference arm was native coronary artery PCI; RR >1 indicates higher risk with SVG PCI, and MD >0 indicates a larger value in the SVG PCI arm.

Supplementary Table S1. Complete leave-one-out sensitivity analyses for all pooled outcomes.

Outcome	Full-model estimate	k	Sensitivity verdict	Study omitted	Re-pooled estimate (95% CI)	I^2 (%)	P value
MACE	1.30 (1.05, 1.60)	16	FRAGILE	Redfors et al., 2017	1.18 (1.00, 1.39)	69.5	0.046
				Boddu et al., 2023	1.26 (1.01, 1.58)	86.7	0.041
				Liu et al., 2019	1.30 (1.04, 1.62)	87.0	0.020
				Tejada et al., 2008	1.31 (1.06, 1.63)	87.0	0.014
				Wei et al., 2013	1.29 (1.03, 1.60)	87.0	0.025
				de Winter et al., 2026	1.38 (1.12, 1.71)	85.3	0.003
				Cohen et al., 2024	1.33 (1.06, 1.67)	86.6	0.013
				Beerkens et al., 2024	1.29 (0.98, 1.68)	86.5	0.070
				Yang et al., 2023	1.29 (1.02, 1.64)	87.0	0.033
				Liu et al., 2023	1.30 (1.04, 1.63)	87.0	0.024
				Beerkens et al., 2021	1.34 (1.07, 1.68)	81.5	0.012
				Alidoosti et al., 2011	1.30 (1.05, 1.62)	87.0	0.016

Outcome	Full-model estimate	k	Sensitivity verdict	Study omitted	Re-pooled estimate (95% CI)	I² (%)	P value
				Bundhoo et al., 2011	1.26 (1.01, 1.57)	86.7	0.037
				D'Ascenzo et al., 2010	1.32 (1.05, 1.64)	86.9	0.015
				Eid-Lidt et al., 2009	1.28 (1.03, 1.60)	87.0	0.028
				Varghese et al., 2008	1.29 (1.04, 1.60)	87.0	0.020
All-cause mortality	1.47 (1.19, 1.81)	16	robust	Redfors et al., 2017	1.39 (1.12, 1.71)	89.9	0.002
				Boddu et al., 2023	1.45 (1.17, 1.79)	90.9	<0.001
				Garcia-Tejada et al., 2009	1.43 (1.16, 1.76)	90.7	<0.001
				Mavroudis et al., 2016	1.47 (1.18, 1.83)	91.0	<0.001
				Wei et al., 2013	1.43 (1.17, 1.77)	90.7	<0.001
				de Winter et al., 2026	1.50 (1.21, 1.85)	90.9	<0.001
				Cohen et al., 2024	1.50 (1.29, 1.74)	72.0	<0.001
				Beerkens et al., 2024	1.48 (1.18, 1.87)	90.5	<0.001
				Yang et al., 2023	1.49 (1.20, 1.86)	91.0	<0.001
				Liu et al., 2023	1.39 (1.13, 1.71)	90.3	0.002
				Broughton et al., 2022	1.54 (1.20, 1.99)	91.0	<0.001
				Beerkens et al., 2021	1.54 (1.23, 1.92)	90.8	<0.001
				Brilakis et al., 2016	1.51 (1.18, 1.94)	89.3	0.001
				Yamaji et al., 2013	1.46 (1.17, 1.82)	90.9	<0.001
				D'Ascenzo et al., 2010	1.45 (1.17, 1.79)	90.9	<0.001
				Varghese et al., 2008	1.46 (1.18, 1.80)	90.9	<0.001
MI	1.64 (1.31, 2.05)	16	robust	Redfors et al., 2017	1.51 (1.23, 1.86)	47.3	<0.001
				Boddu et al., 2023	1.64 (1.29, 2.09)	67.4	<0.001
				Garcia-Tejada et al., 2009	1.66 (1.32, 2.07)	66.5	<0.001
				Liu et al., 2019	1.64 (1.30, 2.07)	67.5	<0.001
				Wei et al., 2013	1.65 (1.31, 2.07)	67.3	<0.001

Outcome	Full-model estimate	k	Sensitivity verdict	Study omitted	Re-pooled estimate (95% CI)	I² (%)	P value
				de Winter et al., 2026	1.78 (1.47, 2.17)	51.5	<0.001
				Cohen et al., 2024	1.65 (1.30, 2.10)	67.3	<0.001
				Beerkens et al., 2024	1.71 (1.36, 2.16)	64.0	<0.001
				Yang et al., 2023	1.60 (1.26, 2.03)	66.9	<0.001
				Liu et al., 2023	1.60 (1.26, 2.01)	66.5	<0.001
				Beerkens et al., 2021	1.67 (1.29, 2.16)	65.0	<0.001
				Brilakis et al., 2016	1.62 (1.20, 2.18)	67.5	0.002
				Yamaji et al., 2013	1.58 (1.25, 1.98)	65.3	<0.001
				Alidoosti et al., 2011	1.63 (1.29, 2.04)	67.3	<0.001
				Bundhoo et al., 2011	1.63 (1.30, 2.05)	67.1	<0.001
				D'Ascenzo et al., 2010	1.65 (1.32, 2.08)	67.2	<0.001
In-stent restenosis	1.71 (1.19, 2.44)	10	robust	Redfors et al., 2017	1.50 (1.07, 2.12)	67.2	0.020
				Boddu et al., 2023	1.60 (1.10, 2.32)	73.0	0.013
				Liu et al., 2019	1.64 (1.15, 2.34)	74.2	0.007
				Mavroudis et al., 2016	1.53 (1.08, 2.16)	69.6	0.017
				Cohen et al., 2024	1.94 (1.25, 3.02)	71.8	0.003
				Liu et al., 2023	1.62 (1.13, 2.32)	73.8	0.009
				Abdelrahman et al., 2022	1.91 (1.32, 2.76)	72.3	<0.001
				Beerkens et al., 2021	1.88 (1.17, 3.04)	76.0	0.009
				Yamaji et al., 2013	1.90 (1.24, 2.90)	75.0	0.003
				D'Ascenzo et al., 2010	1.71 (1.18, 2.47)	76.0	0.004
Cardiac death	1.61 (0.78, 3.32)	7	n/s (full model)	Redfors et al., 2017	1.26 (0.54, 2.93)	66.1	0.597
				Liu et al., 2019	1.69 (0.77, 3.73)	79.7	0.190
				Liu et al., 2023	1.46 (0.62, 3.45)	80.4	0.389
				Yamaji et al., 2013	1.45 (0.50, 4.22)	80.3	0.492

Outcome	Full-model estimate	k	Sensitivity verdict	Study omitted	Re-pooled estimate (95% CI)	I² (%)	P value
				Xanthopoulou et al., 2011	2.69 (1.92, 3.75)	10.8	<0.001
				Bundhoo et al., 2011	1.52 (0.71, 3.24)	80.3	0.280
				D'Ascenzo et al., 2010	1.67 (0.79, 3.54)	80.0	0.182
Stroke	0.94 (0.45, 1.98)	4	n/s (full model)	Cohen et al., 2024	1.09 (0.48, 2.47)	52.6	0.845
				Yang et al., 2023	1.31 (0.70, 2.47)	17.2	0.398
				Liu et al., 2023	0.80 (0.28, 2.24)	64.4	0.664
				Yamaji et al., 2013	0.66 (0.31, 1.40)	4.3	0.278
Bleeding	1.07 (0.81, 1.42)	3	n/s (full model)	Redfors et al., 2017	0.65 (0.12, 3.51)	42.8	0.620
				Liu et al., 2019	1.09 (0.83, 1.45)	0.0	0.529
				Liu et al., 2023	0.68 (0.13, 3.62)	47.1	0.649
TLR	1.70 (1.16, 2.47)	7	FRAGILE	Boddu et al., 2023	1.62 (1.07, 2.45)	70.7	0.023
				Liu et al., 2019	1.67 (1.09, 2.55)	71.3	0.017
				Mavroudis et al., 2016	1.54 (1.04, 2.29)	66.8	0.030
				de Winter et al., 2026	1.95 (1.63, 2.34)	8.0	<0.001
				Beerkens et al., 2024	1.56 (0.83, 2.95)	71.2	0.171
				Yamaji et al., 2013	1.52 (0.83, 2.76)	69.2	0.172
				Alidoosti et al., 2011	1.76 (1.22, 2.54)	66.8	0.002
TVR	1.68 (1.00, 2.80)	14	FRAGILE	Redfors et al., 2017	1.61 (0.90, 2.90)	97.5	0.110
				Boddu et al., 2023	1.66 (0.97, 2.83)	97.5	0.062
				Liu et al., 2019	1.69 (0.99, 2.89)	97.5	0.054
				Mavroudis et al., 2016	1.59 (0.91, 2.76)	97.5	0.100
				Wei et al., 2013	1.74 (1.02, 2.96)	97.5	0.041
				de Winter et al., 2026	1.84 (1.08, 3.12)	97.4	0.024
				Cohen et al., 2024	1.46 (0.87, 2.46)	97.2	0.157
				Beerkens et al., 2024	1.79 (1.05, 3.06)	97.3	0.033

Outcome	Full-model estimate	k	Sensitivity verdict	Study omitted	Re-pooled estimate (95% CI)	I² (%)	P value
				Yang et al., 2023	1.73 (1.00, 2.99)	97.4	0.049
				Broughton et al., 2022	1.54 (1.00, 2.36)	94.0	0.049
				Beerkens et al., 2021	1.81 (1.11, 2.93)	95.7	0.017
				Alidoosti et al., 2011	1.76 (1.05, 2.97)	97.5	0.032
				Bundhoo et al., 2011	1.61 (0.95, 2.75)	97.5	0.079
				Varghese et al., 2008	1.70 (0.97, 2.97)	97.4	0.063
Lesion length (mm)	0.16 (-1.41, 1.73)	7	n/s (full model)	Garcia-Tejada et al., 2009	-0.06 (-1.69, 1.56)	97.5	0.940
				Liu et al., 2019	0.51 (-1.06, 2.08)	97.4	0.522
				de Winter et al., 2026	0.66 (-0.95, 2.26)	97.4	0.421
				Brilakis et al., 2016	-0.30 (-3.35, 2.75)	79.7	0.849
				Zając et al., 2016	-0.36 (-2.00, 1.28)	97.4	0.666
				Alidoosti et al., 2011	0.37 (-1.33, 2.07)	97.4	0.672
				Brilakis et al., 2011	-0.72 (-3.51, 2.07)	75.0	0.612
Number of stents	-0.16 (-0.47, 0.16)	8	n/s (full model)	Garcia-Tejada et al., 2009	-0.23 (-0.57, 0.11)	93.8	0.186
				Liu et al., 2019	-0.15 (-0.50, 0.21)	94.5	0.417
				Wei et al., 2013	-0.19 (-0.54, 0.16)	94.5	0.283
				de Winter et al., 2026	0.04 (-0.13, 0.20)	71.8	0.673
				Yamaji et al., 2013	-0.16 (-0.56, 0.25)	94.4	0.445
				Alidoosti et al., 2011	-0.20 (-0.58, 0.17)	94.0	0.286
				D'Ascenzo et al., 2010	-0.15 (-0.49, 0.19)	94.5	0.381
				Varghese et al., 2008	-0.20 (-0.58, 0.18)	94.2	0.313
Stent diameter (mm)	0.29 (0.22, 0.36)	6	robust	Garcia-Tejada et al., 2009	0.31 (0.24, 0.38)	51.6	<0.001
				Wei et al., 2013	0.29 (0.21, 0.37)	66.7	<0.001
				Cohen et al., 2024	0.29 (0.18, 0.40)	65.3	<0.001

Outcome	Full-model estimate	k	Sensitivity verdict	Study omitted	Re-pooled estimate (95% CI)	I² (%)	P value
				Abdelrahman et al., 2022	0.27 (0.20, 0.34)	52.5	<0.001
				Yamaji et al., 2013	0.31 (0.23, 0.40)	51.0	<0.001
				Alidoosti et al., 2011	0.28 (0.20, 0.35)	60.5	<0.001
Stent length (mm)	-7.17 (-11.66, -2.68)	8	FRAGILE	de Winter et al., 2026	-2.14 (-4.67, 0.40)	88.6	0.099
				Cohen et al., 2024	-9.19 (-15.95, -2.43)	96.6	0.008
				Yang et al., 2023	-6.78 (-11.53, -2.03)	96.5	0.005
				Abdelrahman et al., 2022	-8.40 (-13.98, -2.81)	97.0	0.003
				Zając et al., 2016	-8.97 (-13.84, -4.10)	97.0	<0.001
				Yamaji et al., 2013	-7.72 (-12.74, -2.70)	97.0	0.003
				Alidoosti et al., 2011	-8.82 (-14.62, -3.01)	97.1	0.003
				D'Ascenzo et al., 2010	-7.41 (-12.17, -2.65)	97.1	0.002

Note: CI = confidence interval; I² = Higgins-Thompson inconsistency statistic; ISR = in-stent restenosis; k = number of contributing studies; MACE = major adverse cardiovascular events; MD = mean difference (SVG minus native); MI = myocardial infarction; RR = risk ratio (SVG vs native, native = reference); SVG = saphenous vein graft; TLR = target lesion revascularization; TVR = target vessel revascularization. "Robust" indicates statistical significance in the full model and every single-study omission; "FRAGILE" indicates significance in the full model but loss of significance after at least one omission; "n/s (full model)" indicates non-significance in the full model.

Supplementary Table S2. Pooled estimates with and without the randomized PROCTOR trial (de Winter et al., 2026).

Outcome	Measure	PROCTOR contributes	k with	With PROCTOR (95% CI)	P	I² (%)	k without	Without PROCTOR (95% CI)	P	I² (%)
MACE	RR	Yes	16	1.30 (1.05, 1.60)	0.017	86.1	15	1.38 (1.12, 1.71)	0.003	85.3
All-cause mortality	RR	Yes	16	1.47 (1.19, 1.81)	<0.001	90.3	15	1.50 (1.21, 1.85)	<0.001	90.9
MI	RR	Yes	16	1.64 (1.31, 2.05)	<0.001	65.2	15	1.78 (1.47, 2.17)	<0.001	51.5
In-stent restenosis	RR	No	10	1.71 (1.19, 2.44)	0.004	73.1	10	1.71 (1.19, 2.44)	0.004	73.1
Cardiac death	RR	No	7	1.61 (0.78, 3.32)	0.202	76.5	7	1.61 (0.78, 3.32)	0.202	76.5
Stroke	RR	No	4	0.94 (0.45, 1.98)	0.880	47.5	4	0.94 (0.45, 1.98)	0.880	47.5
Bleeding	RR	No	3	1.07 (0.81, 1.42)	0.620	0.0	3	1.07 (0.81, 1.42)	0.620	0.0

Outcome	Measure	PROCTOR contributes	k with	With PROCTOR (95% CI)	P	I² (%)	k without	Without PROCTOR (95% CI)	P	I² (%)
TLR	RR	Yes	7	1.70 (1.16, 2.47)	0.006	65.5	6	1.95 (1.63, 2.34)	<0.001	8.0
TVR	RR	Yes	14	1.68 (1.00, 2.80)	0.049	97.3	13	1.84 (1.08, 3.12)	0.024	97.4
Lesion length (mm)	MD	Yes	7	0.16 (-1.41, 1.73)	0.843	97.0	6	0.66 (-0.95, 2.26)	0.421	97.4
Number of stents	MD	Yes	8	-0.16 (-0.47, 0.16)	0.336	93.6	7	0.04 (-0.13, 0.20)	0.673	71.8
Stent diameter (mm)	MD	No	6	0.29 (0.22, 0.36)	<0.001	58.4	6	0.29 (0.22, 0.36)	<0.001	58.4
Stent length (mm)	MD	Yes	8	-7.17 (-11.66, -2.68)	0.002	96.6	7	-2.14 (-4.67, 0.40)	0.099	88.6

Note: Primary pooled estimates include PROCTOR; the sensitivity set excludes it. Outcomes to which PROCTOR does not contribute are shown unchanged for completeness. PROCTOR contributes to MACE, all-cause mortality, MI, TLR, TVR, lesion length, number of stents, and stent length. Abbreviations as in Supplementary Table S1.

Supplementary Table S3. 95% prediction intervals and Hartung-Knapp-Sidik-Jonkman sensitivity confidence intervals for all pooled outcomes.

Outcome	Measure	k	Pooled estimate (DL, 95% CI)	95% prediction interval	HKSJ 95% CI	HKSJ P value
MACE	RR	16	1.30 (1.05, 1.60)	(0.60, 2.80)	(1.03, 1.63)	0.031
All-cause mortality	RR	16	1.47 (1.19, 1.81)	(0.71, 3.02)	(1.09, 1.97)	0.014
MI	RR	16	1.64 (1.31, 2.05)	(0.83, 3.25)	(1.26, 2.13)	0.001
In-stent restenosis	RR	10	1.71 (1.19, 2.44)	(0.59, 4.95)	(1.03, 2.83)	0.041
Cardiac death	RR	7	1.61 (0.78, 3.32)	(0.19, 13.71)	(0.65, 3.98)	0.249
Stroke	RR	4	0.94 (0.45, 1.98)	(0.06, 14.90)	(0.28, 3.15)	0.889
Bleeding	RR	3	1.07 (0.81, 1.42)	(0.18, 6.55)	(0.58, 1.98)	0.669
TLR	RR	7	1.70 (1.16, 2.47)	(0.61, 4.72)	(0.90, 3.20)	0.088
TVR	RR	14	1.68 (1.00, 2.80)	(0.21, 13.14)	(0.95, 2.95)	0.070
Lesion length (mm)	MD	7	0.16 (-1.41, 1.73)	(-4.14, 4.46)	(-3.14, 3.46)	0.910
Number of stents	MD	8	-0.16 (-0.47, 0.16)	(-1.28, 0.97)	(-0.61, 0.30)	0.445
Stent diameter (mm)	MD	6	0.29 (0.22, 0.36)	(0.09, 0.49)	(0.19, 0.40)	<0.001
Stent length (mm)	MD	8	-7.17 (-11.66, -2.68)	(-23.01, 8.67)	(-21.55, 7.22)	0.277

Note: DL = DerSimonian-Laird; HKSJ = Hartung-Knapp-Sidik-Jonkman; CI = confidence interval; PI = prediction interval (expected range of the true effect in a comparable future setting). HKSJ CIs use the t distribution with k−1 degrees of freedom. For MACE, cardiac death, stroke, bleeding, and TVR, an ad hoc variance

correction was applied (HKSJ variance floored at the DL variance) to avoid spuriously narrow intervals when k was small. Other abbreviations as in Supplementary Table S1.

Supplementary Table S4. Exploratory drug-eluting stent–predominant subgroup analysis.

Outcome	k	RR (95% CI)	P value	I^2 (%)
MACE	6	1.23 (1.02, 1.49)	0.035	21.5
All-cause mortality	6	1.42 (0.90, 2.23)	0.133	95.5
MI	6	1.72 (1.55, 1.91)	<0.001	0.0
In-stent restenosis	5	1.90 (0.85, 4.22)	0.116	78.2
Cardiac death	3	2.03 (0.90, 4.60)	0.089	0.0
Stroke	3	0.66 (0.31, 1.40)	0.278	4.3
Bleeding	2	0.65 (0.12, 3.51)	0.620	42.8
TLR	2	2.58 (1.27, 5.21)	0.008	18.1
TVR	6	2.37 (1.21, 4.64)	0.012	93.9

Note: Studies were classified as DES-predominant when the native-arm DES proportion was ≥0.5 at the study level. Of studies with stent-type data, 10 were DES-predominant and 2 were BMS/mixed; **the remaining 13 included studies (12 of which contributed binary outcomes) could not be classified**. The BMS/mixed stratum had fewer than 2 poolable studies for every outcome and was therefore not pooled. Results are exploratory and descriptive; between-group comparisons were underpowered given the small BMS/mixed stratum. DES = drug-eluting stent; BMS = bare-metal stent; other abbreviations as in Supplementary Table S1.

Supplementary Table S5. ROBINS-I risk-of-bias assessment of the 24 nonrandomized studies, by domain and overall.

Study [ref]	D1 Confounding	D2 Selection of participants	D3 Classification of interventions	D4 Deviations from intended interventions	D5 Missing data	D6 Measurement of outcomes	D7 Selection of reported result	Overall
Redfors et al., 2017 [52]	Serious	Low	Low	Low	Low	Low	Moderate	Serious
Boddu et al., 2023 [51]	Serious	Low	Low	Low	Moderate	Moderate	Moderate	Serious
Liu et al., 2019 [43]	Serious	Low	Low	Low	Low	Moderate	Moderate	Serious
Tejada et al., 2008 [49]	Serious	Low	Low	Low	Low	Moderate	Moderate	Serious
García-Tejada et al., 2009 [41]	Serious	Low	Low	Low	Low	Moderate	Moderate	Serious
Wei et al., 2013 [53]	Serious	Low	Low	Low	Low	Moderate	Moderate	Serious
Cohen et al., 2024 [47]	Serious	Low	Low	Low	Low	Moderate	Moderate	Serious
Beerkens et al., 2024 [46]	Serious	Low	Low	Low	Moderate	Moderate	Moderate	Serious
Yang et al., 2023 [45]	Serious	Low	Low	Low	Low	Moderate	Moderate	Serious
Liu et al., 2023 [50]	Serious	Low	Low	Low	Low	Low	Moderate	Serious
Beerkens et al., 2021 [44]	Serious	Low	Moderate	Low	Moderate	Low	Moderate	Serious
Alidoosti et al., 2011 [38]	Serious	Low	Low	Low	Low	Moderate	Moderate	Serious
Bundhoo et al., 2011 [37]	Serious	Low	Low	Low	Serious	Moderate	Moderate	Serious

Study [ref]	D1 Confounding	D2 Selection of participants	D3 Classification of interventions	D4 Deviations from intended interventions	D5 Missing data	D6 Measurement of outcomes	D7 Selection of reported result	Overall
D'Ascenzo et al., 2010 [34]	Serious	Low	Low	Low	No information	Moderate	Moderate	Serious
Eid-Lidt et al., 2009 [33]	Serious	Low	Low	Low	No information	Moderate	Moderate	Serious
Varghese et al., 2008 [35]	Serious	Moderate	Low	Low	Low	Moderate	Moderate	Serious
Mavroudis et al., 2016 [42]	Serious	Low	Low	Low	Low	Moderate	Moderate	Serious
Broughton et al., 2022 [54]	Serious	Low	Low	Low	Low	Moderate	Moderate	Serious
Brilakis et al., 2016 [5]	Serious	Low	Low	Low	Moderate	Moderate	Moderate	Serious
Brilakis et al., 2011 [36]	Serious	Low	Low	Low	Low	Moderate	Moderate	Serious
Yamaji et al., 2013 [40]	Serious	Low	Moderate	Low	Low	Low	Moderate	Serious
Abdelrahman et al., 2022 [32]	Serious	Low	Moderate	Low	Moderate	Moderate	Moderate	Serious
Xanthopoulou et al., 2011 [39]	Serious	Low	Low	Low	Low	Moderate	Moderate	Serious
Zając et al., 2016 [48]	Serious	Low	Low	Low	No information	Moderate	Moderate	Serious

ROBINS-I = Risk Of Bias In Non-randomized Studies of Interventions (2016 version); judgments are low, moderate, serious, critical, or no information. Target effect: initiating native coronary artery PCI rather than SVG PCI at the index procedure; prespecified confounders are listed in Section 2.4. Judgments refer to the unadjusted event counts used in this meta-analysis; where judgments differed across outcomes within a study, the most severe judgment is shown. The overall judgment equals the most severe domain judgment. CABG = coronary artery bypass grafting; MI = myocardial infarction; PCI = percutaneous coronary intervention; SVG = saphenous vein graft; TLR/TVR = target lesion/vessel revascularization.

Supplementary Table S5 (continued). Basis for the ROBINS-I judgments.

Study	Basis for judgments
Redfors et al., 2017	Retained because the SVG PCI arm (n = 405, all with prior CABG) met the eligibility criteria and the study provides contemporary, prospectively collected, adjudicated outcomes from a large multicenter registry. The comparator arm in the main analysis was all non-SVG PCI in ADAPT-DES (n = 8,177), of whom 13% had prior CABG, so prior CABG status differs between arms in the unadjusted counts pooled here (D1 serious). Confounding was judged serious rather than critical because the authors reported sensitivity analyses restricted to patients with prior CABG, in which SVG PCI remained associated with higher risk (MACE: multivariable-adjusted HR 2.38, 95% CI 1.69–3.34; propensity-matched HR 2.35, 95% CI 1.53–3.61), indicating that the direction of the association is not explained by the composition of the comparator arm. Its influence on the pooled results was examined in the leave-one-out analysis (Supplementary Table S1): after its omission, MACE (RR 1.18, 95% CI 1.00–1.39), all-cause mortality (RR 1.39, 95% CI 1.12–1.71), and in-stent restenosis (RR 1.50, 95% CI 1.07–2.12) remained significant. Prospective scheduled follow-up with adjudicated death, MI, and stent thrombosis (D5, D6 low); post hoc sub-analysis without a registered protocol (D7).
Boddu et al., 2023	Retrospective single-center cohort; pooled estimate based on unadjusted event counts (D1). 14 of 219 patients (6.4%) lost to follow-up (D5). Outcomes from records, telephone or mailed questionnaire without blinded adjudication (D6). No protocol (D7).
Liu et al., 2019	Retrospective single-center cohort of diabetic patients; unadjusted counts (D1). Only 3 of 171 (1.8%) excluded for missing follow-up (D5 low). Events from clinical follow-up without independent adjudication (D6). No protocol (D7).
Tejada et al., 2008	Consecutive single-center cohort (Hospital 12 de Octubre, Madrid); unadjusted counts (D1). Complete 1-year follow-up (D5 low). MI by routine CK-MB; repeat revascularization clinically driven and unblinded (D6). No protocol (D7). Same center and author group as García-Tejada et al., 2009.
García-Tejada et al., 2009	Consecutive single-center cohort; unadjusted counts (D1). Follow-up available for all patients (D5 low). Clinically driven, unblinded revascularization; no adjudication (D6). No protocol (D7).
Wei et al., 2013	Liu W et al., Chin Med J 2013 (labelled Wei et al. in Tables 1–2 and the figures). Consecutive STEMI patients with prior CABG from a single-center prospective PCI registry (Beijing); unadjusted counts (D1). All patients completed follow-up (D5 low). Follow-up by clinic or telephone; angiography not routine; no adjudication reported (D6). No protocol (D7).
Cohen et al., 2024	Prospective multicenter registry (Melbourne Interventional Group); unadjusted counts (D1). Long-term mortality by National Death Index linkage (99.4% matched) (D5 low). 30-day events verified and audited by data managers but not blinded to target vessel (D6). No protocol (D7).
Beerkens et al., 2024	Nationwide prospective registry (Netherlands Heart Registration); unadjusted counts (D1). TLR collected only from 2019 onwards and some variables missing (D5). Endpoints audited by a nursing specialist but not blinded (D6). No protocol (D7).
Yang et al., 2023	Retrospective single-center cohort (Beijing Anzhen Hospital) of consecutive patients; IPTW-adjusted analyses reported, but unadjusted counts pooled here (D1). 55 of 1,469 eligible patients (3.7%) lost to follow-up and excluded (D5 low). Follow-up by clinic visit or telephone; unblinded ascertainment (D6). No protocol (D7).
Liu et al., 2023	Prospective single-center cohort (Fuwai Hospital); unadjusted counts (D1). Follow-up complete for all patients (D5 low). Telephone follow-up overseen by independent physicians with blinded questionnaires (D6 low). No protocol (D7).
Beerkens et al., 2021	Single-center database analyzed retrospectively; unadjusted counts (D1). Patients with combined native and graft PCI were assigned to the graft group (D3). 1-year telephone and record follow-up; completeness not reported (D5). Events adjudicated by a clinical events committee (D6 low). No protocol (D7).
Alidoosti et al., 2011	Consecutive single-center registry (Tehran Heart Center); unadjusted counts (D1). About 5% lost to follow-up (D5 low). MI by CK-MB; TVR clinically driven without routine angiography or adjudication (D6). No protocol (D7).
Bundhoo et al., 2011	Two-center retrospective analysis; unadjusted counts (D1). 29 of 190 consecutive patients (15%) excluded because follow-up data were incomplete, without analysis of the excluded patients (D5 serious). Periprocedural MI not measured; repeat PCI clinically driven (D6). No protocol (D7).
D'Ascenzo et al., 2010	Retrospective single-center cohort; unadjusted counts (D1). Long-term follow-up by telephone or clinic visits; completeness not reported (D5 no information). Unblinded event ascertainment (D6). No protocol (D7).
Eid-Lidt et al., 2009	Retrospective single-center cohort; unadjusted counts (D1). Exclusions (acute MI, total SVG occlusion, SVG <3 months) applied at baseline (D2 low). Completeness of 3-year follow-up not reported (D5 no information). Unblinded event ascertainment (D6). No protocol (D7).

Study	Basis for judgments
Varghese et al., 2008	Retrospective single-center cohort (Dallas VA); unadjusted counts (D1). Patients enrolled in a concurrent randomized trial of stents in SVG lesions were excluded, selectively removing part of the SVG population (D2). Follow-up available for all patients (D5 low). Events from medical records without adjudication (D6). No protocol (D7).
Mavroudis et al., 2016	Retrospective single-center cohort (Royal Free, London); unadjusted counts (D1). Mortality by Office for National Statistics tracking (D5 low). TVR from records, not adjudicated (D6). Analyses mix procedure- and patient-level denominators; no protocol (D7).
Broughton et al., 2022	Retrospective multi-hospital health-system cohort (UPMC); unadjusted counts (D1). Arterial grafts (n = 65) combined with SVGs in the graft group. Mortality from social security and medical records; censoring handled by time-to-event methods (D5 low). Readmissions identified by ICD/CPT codes (D6). No protocol (D7).
Brilakis et al., 2016	National Veterans Affairs registry (VA CART); unadjusted counts (D1). Mortality from VA vital status file, but MI and revascularization outside VA facilities may have been missed (D5). Events from administrative and clinical records (D6). No protocol (D7).
Brilakis et al., 2011	National registry (NCDR CathPCI); unadjusted data (D1). In-hospital data with standard registry definitions and data-quality checks but no core-laboratory angiographic adjudication (D6). No protocol (D7).
Yamaji et al., 2013	Post hoc subgroup analysis of the multicenter j-Cypher registry (Japan), which enrolled consecutive patients receiving sirolimus-eluting stents with follow-up obtained under the registry protocol through 5 years; no differential loss to follow-up between the SVG and native groups was reported (D5 low), and myocardial infarction and stent thrombosis were adjudicated using standardized definitions (D6 low). Multivariable-adjusted hazard ratios were reported, but the unadjusted counts were pooled here (D1 serious). Patients treated in both an SVG and a native coronary artery were analyzed in the SVG group, consistent with the approach of several other included studies (D3 moderate). In the leave-one-out analysis (Supplementary Table S1), its omission did not change the all-cause mortality, MI, or in-stent restenosis results; omission rendered the TLR association non-significant, as reported in Section 3.4. Post hoc analysis without a protocol (D7).
Abdelrahman et al., 2022	Retrospective single-center cohort; unadjusted counts used (D1). Patients treated in both a native vessel and an SVG were assigned to the SVG arm (D3). Authors state not all patients were routinely followed up at 12 months (D5). Unblinded event ascertainment (D6). No protocol (D7).
Xanthopoulou et al., 2011	Retrospective single-center cohort (Patras, Greece) of 221 consecutive patients; unadjusted counts (D1). Patients with both native and graft PCI (7.2%) excluded at baseline. 15 patients (6.8%) with missing follow-up excluded, but a sensitivity analysis assuming those lost had died was performed (D5 low). Follow-up from hospital records and telephone; follow-up angiograms read independently (D6 moderate for clinical events). No protocol (D7).
Zając et al., 2016	Prospective single-center observational study of 78 consecutive post-CABG patients (20 SVG PCI, 29 native PCI, 29 medical therapy). The 50-patient eligibility criterion was applied to the total study population, which this study meets (n = 78; by contrast, Meliga et al., 2007 enrolled 24 patients in total). It contributes only to the procedural outcomes (lesion length and stent length), and its omission did not change either result (lesion length MD −0.36 mm, 95% CI −2.00 to 1.28; stent length MD −8.97 mm, 95% CI −13.84 to −4.10; Supplementary Table S1). Unadjusted data (D1 serious); completeness of 12-month follow-up not reported (D5 no information); unblinded assessment (D6); no protocol (D7).

Supplementary Data

Supplementary Table S6. RoB 2 risk-of-bias assessment of the randomized PROCTOR trial, by domain and outcome.

Outcome (PROCTOR, de Winter et al., 2026 [7])	D1 Randomization process	D2 Deviations from intended interventions	D3 Missing outcome data	D4 Measurement of the outcome	D5 Selection of the reported result	Overall
MACE (1 year)	Low	Low	Low	Some concerns	Some concerns	Some concerns
All-cause mortality (1 year)	Low	Low	Low	Low	Some concerns	Some concerns
Myocardial infarction (1 year)	Low	Low	Low	Some concerns	Some concerns	Some concerns

RoB 2 = Cochrane Risk of Bias 2 tool (22 August 2019 version), effect of assignment to intervention; judgments are low risk, some concerns, or high risk. Some concerns for measurement of MACE and MI reflect the open-label design, clinically driven revascularization within the composite endpoint, and the definition-dependence of periprocedural MI; some concerns for selection of the reported result reflect the need to confirm the dated protocol, statistical analysis plan, and adjudication materials for the one-year analysis.
