# Supplementary material for: Native Coronary Artery Versus Saphenous Vein Graft Percutaneous Coronary Intervention in Patients with Prior Coronary Artery Bypass Grafting: An Updated Systematic Review and Meta-Analysis
Source: J Clin Med. 2026 Nov 6;15(21):8672. doi: 10.3390/jcm15218672 (PMC13764333; doi:10.3390/jcm15218672)
Supplement: Supplementary file 1 [file jcm-15-08672-s001.zip › jcm-4579736-supplementary_PRISMA_final.pdf]

PRISMA 2020 Checklist

Section and Topic	Item #	Checklist item	Location where item is reported
**TITLE**			
Title	1	Identify the report as a systematic review.	**Title (p. 1): identified as "An Updated Systematic Review and Meta-Analysis".**
**ABSTRACT**			
Abstract	2	See the PRISMA 2020 for Abstracts checklist.	**Abstract (p. 1): objective; information sources (PubMed, Embase, Cochrane Library, searched through May 2026); 25 included studies (343,524 patients), 21 contributing to MACE and/or all-cause mortality; pooled RRs with 95% CIs for MACE,**

PRISMA 2020 Checklist

Section and Topic	Item #	Checklist item	Location where item is reported
			mortality, MI, ISR, TLR; analysis without the randomized trial; leave-one-out findings; limitations including prediction intervals and GRADE certainty (low to very low); clinical conclusion.
**INTRODUCTION**			

Section and Topic	Item #	Checklist item	Location where item is reported
Rationale	3	Describe the rationale for the review in the context of existing knowledge.	**Introduction, paragraphs 1–3 (p. 2): SVG failure and repeat revascularization after CABG; observational evidence; confounding by indication; the PROCTOR randomized trial.**
Objectives	4	Provide an explicit statement of the objective(s) or question(s) the review addresses.	**Introduction, final paragraph (p. 3): objective to compare long-term clinical outcomes, repeat revascularization, and procedural characteristics of native coronary artery PCI versus SVG PCI.**

PRISMA 2020 Checklist

Section and Topic	Item #	Checklist item	Location where item is reported
**METHODS**			
Eligibility criteria	5	Specify the inclusion and exclusion criteria for the review and how studies were grouped for the syntheses.	**Methods, Section 2.1 (p. 3): eligibility criteria (prior CABG; native vs. SVG PCI comparison; ≥1 clinical outcome; RCTs and observational studies; ≥50 patients; exclusions), outcome-specific criterion for in-stent restenosis, and grouping of studies for synthesis by outcome.**

PRISMA 2020 Checklist

Section and Topic	Item #	Checklist item	Location where item is reported
Information sources	6	Specify all databases, registers, websites, organisations, reference lists and other sources searched or consulted to identify studies. Specify the date when each source was last searched or consulted.	**Methods, Section 2.1 (p. 3): PubMed, Embase, and the Cochrane Library (CENTRAL), each searched from inception to May 2026; search date also shown in the PRISMA flow diagram (Figure 3, p. 9).**

PRISMA

PRISMA 2020 Checklist

Section and Topic	Item #	Checklist item	Location where item is reported
Search strategy	7	Present the full search strategies for all databases, registers and websites, including any filters and limits used.	**Supplementary Methods S1: full search strategy for PubMed, Embase, and the Cochrane Library, including Boolean operators, MeSH/Emtree terms, truncation, and filters.**
Selection process	8	Specify the methods used to decide whether a study met the inclusion criteria of the review, including how many reviewers screened each record and each report retrieved, whether they worked independently, and if applicable, details of automation tools used in the process.	**Methods, Section 2.1 (p. 3): two reviewers independently screened titles/abstracts and full texts; disagreements resolved by consensus or a third reviewer; no automation tools.**

PRISMA 2020 Checklist

Section and Topic	Item #	Checklist item	Location where item is reported
Data collection process	9	Specify the methods used to collect data from reports, including how many reviewers collected data from each report, whether they worked independently, any processes for obtaining or confirming data from study investigators, and if applicable, details of automation tools used in the process.	**Methods, Section 2.2 (p. 4): two independent reviewers using a standardized form; discrepancies resolved by consensus or a third reviewer; no study investigators contacted.**

PRISMA

PRISMA 2020 Checklist

Section and Topic	Item #	Checklist item	Location where item is reported
Data items	10a	List and define all outcomes for which data were sought. Specify whether all results that were compatible with each outcome domain in each study were sought (e.g. for all measures, time points, analyses), and if not, the methods used to decide which results to collect.	**Methods, Section 2.2 (p. 4): outcomes (MACE, all-cause mortality, MI, ISR, cardiac death, stroke, bleeding, TLR, TVR; stent diameter, stent length, lesion length, number of stents); longest available follow-up sought for each outcome.**

Section and Topic	Item #	Checklist item	Location where item is reported
	10b	List and define all other variables for which data were sought (e.g. participant and intervention characteristics, funding sources). Describe any assumptions made about any missing or unclear information.	**Methods, Section 2.2 (p. 4): study design, sample size, demographics, comorbidities, clinical presentation, prior CABG, lesion/graft and procedural characteristics, stent type, follow-up; missing information treated as unavailable, not imputed.**

Section and Topic	Item #	Checklist item	Location where item is reported
Study risk of bias assessment	11	Specify the methods used to assess risk of bias in the included studies, including details of the tool(s) used, how many reviewers assessed each study and whether they worked independently, and if applicable, details of automation tools used in the process.	**Methods, Section 2.4 (pp. 6–7): RoB 2 (2019 version) for the randomized trial and ROBINS-I (2016 version) for nonrandomized studies, at the level of each outcome estimate; prespecified confounders listed; study-level judgments in Supplementary Tables S5–S6.**

PRISMA

PRISMA 2020 Checklist

Section and Topic	Item #	Checklist item	Location where item is reported
Effect measures	12	Specify for each outcome the effect measure(s) (e.g. risk ratio, mean difference) used in the synthesis or presentation of results.	**Methods, Section 2.3 (p. 4): risk ratios for binary outcomes and mean differences for continuous outcomes, with 95% CIs; all analyses, including the subgroup analysis, use risk ratios.**

Section and Topic	Item #	Checklist item	Location where item is reported
Synthesis methods	13a	Describe the processes used to decide which studies were eligible for each synthesis (e.g. tabulating the study intervention characteristics and comparing against the planned groups for each synthesis (item #5)).	**Methods, Sections 2.1 (p. 3) and 2.2 (p. 4): studies grouped by outcome; included in each synthesis only when they reported the outcome in a form expressible as a common effect measure.**
	13b	Describe any methods required to prepare the data for presentation or synthesis, such as handling of missing summary statistics, or data conversions.	**Methods, Sections 2.2 and 2.3 (p. 4): means and SDs estimated from medians and IQRs by the method of Wan et al.; otherwise outcome not extracted.**

Section and Topic	Item #	Checklist item	Location where item is reported
	13c	Describe any methods used to tabulate or visually display results of individual studies and syntheses.	**Methods, Section 2.2 (p. 4); results displayed in forest plots (Figures 4–15, pp. 10–17), funnel plots (Figures 1–2, pp. 5–6), study-level tables (Tables 1–2, pp. 29–30), and the Central Illustration (Figure 16, p. 18).**

Section and Topic	Item #	Checklist item	Location where item is reported
	13d	Describe any methods used to synthesize results and provide a rationale for the choice(s). If meta-analysis was performed, describe the model(s), method(s) to identify the presence and extent of statistical heterogeneity, and software package(s) used.	**Methods, Section 2.3 (p. 4): DerSimonian–Laird random-effects model; heterogeneity by I^2, Cochran's Q, and τ^2; 95% prediction intervals; Hartung–Knapp–Sidik–Jonkman sensitivity analysis.**

PRISMA

PRISMA 2020 Checklist

Section and Topic	Item #	Checklist item	Location where item is reported
	13e	Describe any methods used to explore possible causes of heterogeneity among study results (e.g. subgroup analysis, meta-regression).	**Methods, Sections 2.2 (p. 4) and 2.3 (p. 6): descriptive exploration of heterogeneity; exploratory DES-predominant subgroup analysis; meta-regression not feasible (reason stated).**

Section and Topic	Item #	Checklist item	Location where item is reported
	13f	Describe any sensitivity analyses conducted to assess robustness of the synthesized results.	**Methods, Section 2.3 (pp. 4–6): leave-one-out analyses for all pooled outcomes with prespecified robustness criteria; analyses repeated after exclusion of the randomized trial; HKSJ-adjusted confidence intervals. Section 2.4 (p. 7) explains why risk-of-bias-restricted analyses were not feasible.**

Section and Topic	Item #	Checklist item	Location where item is reported
Reporting bias assessment	14	Describe any methods used to assess risk of bias due to missing results in a synthesis (arising from reporting biases).	**Methods, Sections 2.2 (p. 4) and 2.3 (p. 4): funnel plots and Egger's test when sufficient studies contributed.**

Section and Topic	Item #	Checklist item	Location where item is reported
Certainty assessment	15	Describe any methods used to assess certainty (or confidence) in the body of evidence for an outcome.	**Methods, Section 2.5 (p. 7): GRADE approach (starting at high certainty with ROBINS-I; domains for rating down and up described); separate ratings for the pooled evidence, the nonrandomized evidence without PROCTOR, and PROCTOR alone.**
**RESULTS**			

PRISMA 2020 Checklist

Section and Topic	Item #	Checklist item	Location where item is reported
Study selection	16a	Describe the results of the search and selection process, from the number of records identified in the search to the number of studies included in the review, ideally using a flow diagram.	**Results, Section 3.1 (p. 8) and PRISMA 2020 flow diagram (Figure 3, p. 9): 387 records identified, 70 duplicates removed, 317 screened, 38 full-text reports assessed, 13 excluded with reasons, 25 studies included.**

Section and Topic	Item #	Checklist item	Location where item is reported
	16b	Cite studies that might appear to meet the inclusion criteria, but which were excluded, and explain why they were excluded.	**Results, Section 3.1 (p. 8): Waldo et al., 2013 (comparator population not post-CABG), Meliga et al., 2007 (fewer than 50 patients), and Ernst et al., 1986 (balloon-angioplasty restenosis, not comparable to in-stent restenosis) excluded at full-text review; remaining exclusions summarized by reason in Figure 3.**

PRISMA

PRISMA 2020 Checklist

Section and Topic	Item #	Checklist item	Location where item is reported
Study characteristics	17	Cite each included study and present its characteristics.	**Results, Section 3.1 (p. 8); Tables 1–2 (pp. 29–30): each included study cited (references 5, 7, and 32–54) with design and setting; contributing studies and participants per outcome in Table 3 (p. 31).**

Section and Topic	Item #	Checklist item	Location where item is reported
Risk of bias in studies	18	Present assessments of risk of bias for each included study.	**Supplementary Tables S5–S6: ROBINS-I judgments by domain and overall for each of the 24 nonrandomized studies, with the basis for each judgment (Table S5), and RoB 2 judgments by domain for the PROCTOR trial (Table S6); summarized in Results, Section 3.6, and incorporated in GRADE Tables 3–4.**

Section and Topic	Item #	Checklist item	Location where item is reported
Results of individual studies	19	For all outcomes, present, for each study: (a) summary statistics for each group (where appropriate) and (b) an effect estimate and its precision (e.g. confidence/credible interval), ideally using structured tables or plots.	**Results; Figures 4–15 (pp. 10–17); Tables 1–2 (pp. 29–30): study-level effect estimates and 95% CIs for every pooled outcome.**

Section and Topic	Item #	Checklist item	Location where item is reported
Results of syntheses	20a	For each synthesis, briefly summarise the characteristics and risk of bias among contributing studies.	**Results, Sections 3.1 (p. 8) and 3.6 (pp. 19–20): study designs, baseline comparability, and risk of bias of contributing studies.**

Section and Topic	Item #	Checklist item	Location where item is reported
	20b	Present results of all statistical syntheses conducted. If meta-analysis was done, present for each the summary estimate and its precision (e.g. confidence/credible interval) and measures of statistical heterogeneity. If comparing groups, describe the direction of the effect.	**Results, Sections 3.2–3.3 (pp. 10–15): pooled RRs/MDs with 95% CIs, I^2, τ^2, and 95% prediction intervals for all outcomes (e.g., MACE RR 1.30, 95% CI 1.05–1.60, I^2 = 86%); Supplementary Table S3.**

Section and Topic	Item #	Checklist item	Location where item is reported
	20c	Present results of all investigations of possible causes of heterogeneity among study results.	**Results, Section 3.5 (p. 19); Supplementary Table S4: DES-predominant subgroup in risk ratios (12 of 25 studies with stent-type data: 10 DES-predominant, 2 BMS/mixed).**

Section and Topic	Item #	Checklist item	Location where item is reported
	20d	Present results of all sensitivity analyses conducted to assess the robustness of the synthesized results.	**Results, Section 3.4 (pp. 18–19); Supplementary Tables S1–S3: leave-one-out results (mortality, MI, ISR, stent diameter robust; MACE, TLR, TVR, stent length fragile); analyses without PROCTOR; HKSJ-adjusted intervals (Section 3.3, p. 14).**

Section and Topic	Item #	Checklist item	Location where item is reported
Reporting biases	21	Present assessments of risk of bias due to missing results (arising from reporting biases) for each synthesis assessed.	**Results, Section 3.3 (p. 14); Figures 1–2 (pp. 5–6): funnel plots and Egger's test for MACE and all-cause mortality.**

Section and Topic	Item #	Checklist item	Location where item is reported
Certainty of evidence	22	Present assessments of certainty (or confidence) in the body of evidence for each outcome assessed.	**Results, Section 3.6 (p. 20); Tables 3–4 (pp. 31–33): GRADE certainty for each clinical outcome (very low for all pooled outcomes; low for MI and TLR without PROCTOR; low to very low for PROCTOR alone).**
**DISCUSSION**			

PRISMA 2020 Checklist

Section and Topic	Item #	Checklist item	Location where item is reported
Discussion	23a	Provide a general interpretation of the results in the context of other evidence.	**Discussion, Sections 4.1–4.3 (pp. 20–21): interpretation in the context of prior observational evidence and the PROCTOR trial.**

Section and Topic	Item #	Checklist item	Location where item is reported
	23b	Discuss any limitations of the evidence included in the review.	**Discussion, Section 4.6 (pp. 23–24): observational design and confounding, heterogeneity, robustness of estimates, incomplete reporting, low to very low certainty.**

Section and Topic	Item #	Checklist item	Location where item is reported
	23c	Discuss any limitations of the review processes used.	**Discussion, Section 4.6 (p. 24): reliance on aggregate data, incomplete covariate reporting, no meta-regression or individual-patient-data analysis.**

Section and Topic	Item #	Checklist item	Location where item is reported
	23d	Discuss implications of the results for practice, policy, and future research.	**Discussion, Sections 4.4–4.5 (p. 22), Future Directions (Section 5, p. 24), and Conclusion (Section 6, p. 25).**
**OTHER INFORMATION**			

PRISMA 2020 Checklist

Section and Topic	Item #	Checklist item	Location where item is reported
Registration and protocol	24a	Provide registration information for the review, including register name and registration number, or state that the review was not registered.	**Methods, Section 2.1 (p. 3): PROSPERO, registration number CRD420261449018.**
	24b	Indicate where the review protocol can be accessed, or state that a protocol was not prepared.	**Methods, Section 2.1 (p. 3): protocol publicly accessible in the PROSPERO database.**

PRISMA 2020 Checklist

Section and Topic	Item #	Checklist item	Location where item is reported
	24c	Describe and explain any amendments to information provided at registration or in the protocol.	**Methods, Section 2.1 (p. 3): post-registration amendments listed (analysis excluding the randomized trial; prediction intervals and HKSJ intervals; GRADE assessment), all introduced during peer review.**
Support	25	Describe sources of financial or non-financial support for the review, and the role of the funders or sponsors in the review.	**Funding statement (p. 25): no external funding.**

PRISMA

PRISMA 2020 Checklist

Section and Topic	Item #	Checklist item	Location where item is reported
Competing interests	26	Declare any competing interests of review authors.	**Conflicts of Interest (p. 25): none declared.**
Availability of data, code and other materials	27	Report which of the following are publicly available and where they can be found: template data collection forms; data extracted from included studies; data used for all analyses; analytic code; any other materials used in the review.	**Data Availability Statement (p. 25): data from published studies; supporting data in the article and Supplementary Materials; extracted data available from the corresponding author on reasonable request.**

From: Page MJ, McKenzie JE, Bossuyt PM, Boutron I, Hoffmann TC, Mulrow CD, et al. The PRISMA 2020 statement: an updated guideline for reporting systematic reviews. BMJ 2021;372:n71. doi: 10.1136/bmj.n71.
This work is licensed under CC BY 4.0. To view a copy of this license, visit https://creativecommons.org/licenses/by/4.0/
